# The Epstein-Barr virus miR-BHRF1-1 targets RNF4 during productive infection to promote the accumulation of SUMO conjugates and the release of infectious virus

**DOI:** 10.1371/journal.ppat.1006338

**Published:** 2017-04-17

**Authors:** Jinlin Li, Simone Callegari, Maria G. Masucci

**Affiliations:** Department of Cell and Molecular Biology, Karolinska Institutet, Stockholm, Sweden; University of Wisconsin-Madison School of Medicine and Public Health, UNITED STATES

## Abstract

Post-translational modification by the Small Ubiquitin-like Modifier (SUMO) regulates a variety of cellular functions, and is hijacked by viruses to remodel the host cell during latent and productive infection. Here we have monitored the activity of the SUMO conjugation machinery in cells productively infected with Epstein-Barr virus (EBV). We found that SUMO2/3 conjugates accumulate during the late phase of the productive virus cycle, and identified several viral proteins as bone fide SUMOylation substrates. Analysis of the mechanism involved in the accumulation of SUMOylated proteins revealed upregulation of several components of the SUMO-conjugation machinery and post-transcriptional downregulation of the SUMO-targeted ubiquitin ligase RNF4. The latter effect was mediated by selective inhibition of RNF4 protein expression by the viral miR-BHRF1-1. Reconstitution of RNF4 in cells expressing an inducible miR-BHRF1-1 sponge or a miR-BHRF1-1 resistant RNF4 was associated with reduced levels of early and late viral proteins and impaired virus release. These findings illustrate a novel strategy for viral interference with the SUMO pathway, and identify the EBV miR-BHRF1-1 and the cellular RNF4 as regulators of the productive virus cycle.

## Author summary

We have investigated the activity of the SUMOylation machinery in cells infected with Epstein-Barr virus (EBV), a human herpesvirus that infects B-lymphocytes and is associated with malignancies. We found that activation of the productive virus cycle is accompanied by accumulation of SUMO conjugates, upregulation of components of the SUMO conjugation machinery, and downregulation of the SUMO-targeted ubiquitin ligase RNF4. The decrease of RNF4 is due to post-transcriptional downregulation by miR-BHRF1-1, a member of the BHRF1 microRNA cluster that is upregulated during productive infection. The effect of miR-BHRF1-1 was confirmed in luciferase reported assays, by mutation of the RNF4 3’UTR seed site, by transfection of a synthetic miR-BHRF1-1 mimic, by ectopic expression of miR-BHRF1-1 and by the reversal of RNF4 downregulation in cells expressing a miR-BHRF1-1 sponge. We also found that several early and late viral proteins are bona fide SUMOylation substrates. Reconstitution of RNF4 in productively infected cells was accompanied by proteasome-dependent degradation of the SUMOylated viral protein and by a significantly reduced virus yield. These findings illustrate a new strategy for viral interference with the SUMO pathway, an unexpected contribution of miR-BHRF1-1 to the productive cycle of EBV and a previously unrecognized role of the RNF4 ligase in the regulation of virus production.

## Introduction

Increasing evidence implicates post-translational modification by the small ubiquitin-like modifiers SUMO1, SUMO2 and SUMO3 in the regulation of a broad variety of cellular functions [1]. Conjugation of the SUMO paralogs, SUMOylation, is a highly dynamic process, with dramatic changes occurring in response to different types of intracellular or exogenous stress, including oxidation, heat shock and hypoxia [2–4].

Similar to ubiquitination, SUMOylation is a multistep process involving an activating enzyme, the SAE1/SAE2 heterodimer [5], a conjugating enzyme, UBC9 [6], and one of several SUMO ligases, including the PIAS (protein inhibitor of activated STATS) family [7–9]. SUMO2 and SUMO3 can polymerize to form polySUMO chains whereas SUMO1 prevalently forms mono conjugates and may serve as terminator of mixed chains [10]. SUMO-specific peptidases, such as the six members of the SENP family, mediate the maturation of SUMO pro-peptides, remove SUMO from conjugates, and depolymerize SUMO chains [11]. In addition, the proteasome-dependent turnover of poly-SUMOylated proteins is modulated by SUMO-targeted ubiquitin ligases (STUbLs), such as the human RNF4 and RNF111 ligases, that recognize their SUMOylated substrates via multiple SUMO interacting motifs (SIMs) [12].

Pathogenic viruses and intracellular bacteria adopt a variety of different strategies to interfere with SUMOylation in order to establish a cellular environment that is favorable to their survival and replication [13]. Bacteria examples include the *Yersinia pestis* virulence protein YopJ that mimics the activity of SENPs to inhibit the MAPK signaling pathway [14], and the listeriolysin-O of *Listeria monocytogenes* that promotes bacterial infection by inducing the proteasomal degradation of UBC9 through a yet unknown mechanism [15]. Different types of viruses exploit or inhibit SUMOylation during different phase of the infection. For example, SUMOylation regulates the function of many immediate-early (IE) and early (E) products of DNA viruses. These are often transcriptional factors, such as the IE1 and IE2 of cytomegalovirus (CMV) [16, 17], E1 and E2 of human papillomavirus (HPV) [18, 19], BZLF1 and BRLF1 of Epstein-Barr virus (EBV) [20, 21], and the K-pZIP of Kaposi's sarcoma associated herpesvirus (KSHV) that also serves as a specific SUMO2/-3 ligase [22]. Some virus structural proteins were shown to be SUMOylated [23]. The early proteins ICP0 of herpes simplex virus (HSV)-1 [24] and K-Rta of KHSV [25] are STUbLs that inhibit antiviral responses by promoting the degradation of the promyelocytic leukemia protein (PML). Viral proteins may modulate the SUMOylation of specific cellular proteins, including the tumor suppressor Rb [26] and the transcriptional co-repressor KAP1 [27, 28], while the avian adenovirus Gaml protein promotes a global impairment of SUMOylation by interfering with the activity of the SAE1/SAE2 heterodimer and by reducing the expression of UBC9 [29, 30].

EBV is a gamma-herpesvirus that establishes latent infection in B-lymphocytes and is associated with lymphoid and epithelial cell malignancies [31]. The switch from latent to productive infection is mediated by products of the immediate early genes BZLF1 and BRLF1 [32]. SUMO1, -2 and -3 modify BZLF1 on Lys12, which regulates its transcriptional activity and is at least partially responsible for the destruction of PML bodies during productive infection [33]. BRLF1 interacts with UBC9, RanBP2 and PIAS1 and is modified by the SUMO paralogs at multiple Lys residues [21, 34, 35]. Interaction with the EBV L2 protein promotes SUMOylation while inhibiting the transcriptional activity of BRLF1 [35]. In addition, the early protein BGLF4 binds SUMO2 through N- and C-terminal SIM motifs. Mutation of the SIMs changes the intracellular localization of BGLF4 and inhibits its capacity to: regulate the SUMOylation of BZLF1, trigger the DNA damage response, and promote virus release [36]. The latent membrane protein (LMP)-1 interacts with UBC9 via its C-terminal activating region (CTAR)-3 [37], and promotes SUMOylation of the interferon regulatory factor IRF7, which limits the transcriptional activity of IRF7 and the activation of innate immune responses [38]. Collectively, these findings point to a pivotal role of SUMOylation in the regulation of both viral and cellular functions during different phases of EBV infection.

In this study, we have monitored the abundance of SUMO conjugates in EBV infected cells entering the productive virus cycle. We found that virus replication is accompanied by a robust accumulation of poly-SUMOylated proteins, which is mediated by transcriptional upregulation of several components of the SUMOylation machinery, and post-transcriptional downregulation of RNF4 due to selective blockade of RNF4 expression by the EBV encoded BHRF1-1 miRNA. Reversal of RNF4 downregulation in productively infected cells expressing a miR-BHRF1-1 sponge or a miR-BHRF1-1 resistant RNF4 was accompanied by reduced accumulation of SUMO conjugates, proteasome-dependent degradation of viral proteins and impaired release of infectious viral particles. Thus, miR-BHRF1-1 promotes virus production by regulating a RNF4-mediated cellular defense against infection.

## Results

### Poly-SUMO conjugates accumulate during productive infection

**Fig 1:**
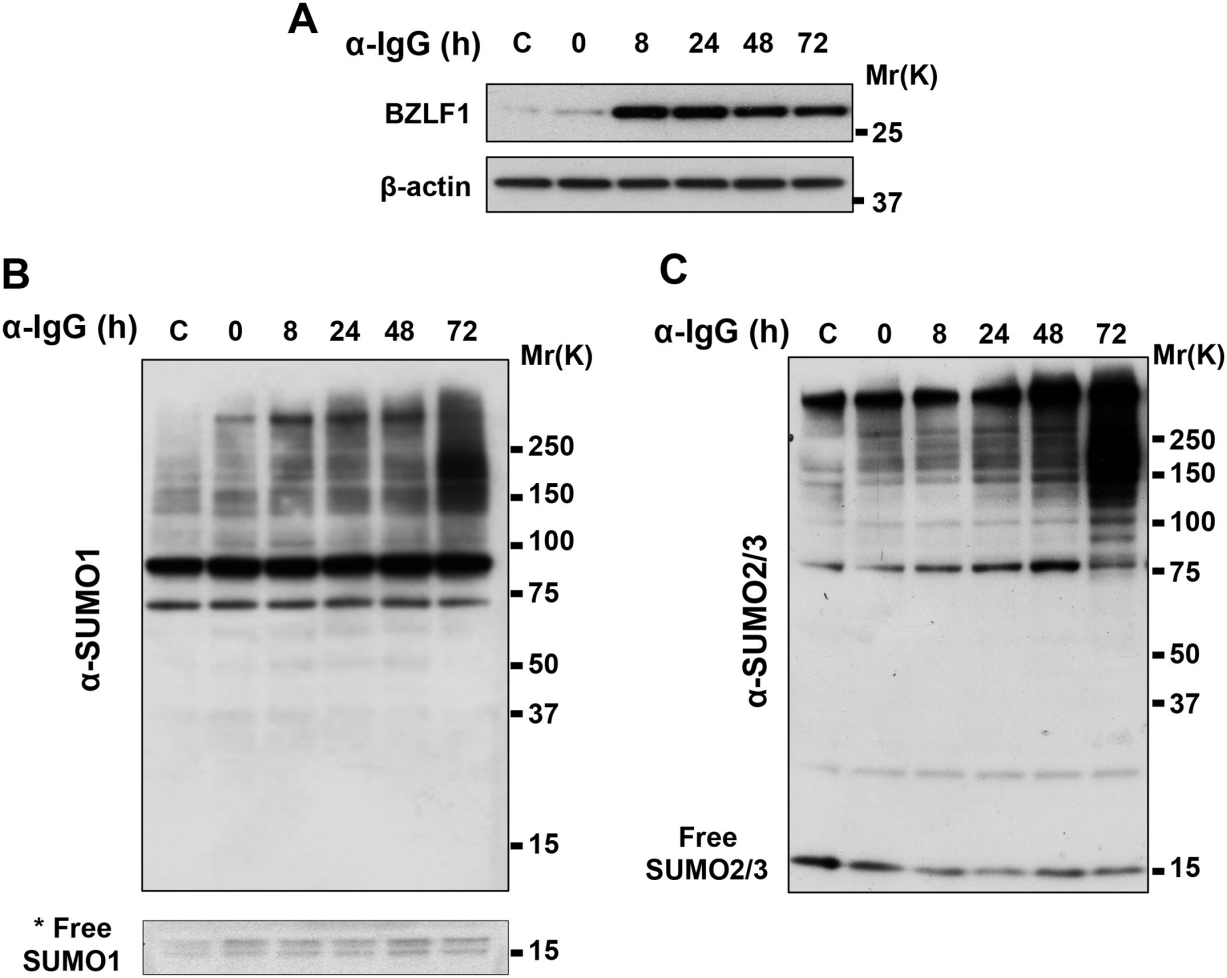
SUMO conjugates accumulate during productive EBV infection. Akata-Bx1 cells were harvested at the indicated times after induction by anti-IgG cross-linking and western blots were probed with antibodies to SUMO1 or SUMO2/3. (**A**) Induction of the productive cycle was confirmed in western blots probes with antibodies to the EBV transactivator BZLF1. Representative western blots illustrating the progressive accumulation of high molecular weight polypeptides reacting with antibodies to SUMO1 (**B**) and SUMO2/3 (**C**) antibodies; β-actin was used as loading control. * a longer exposure was required to visualize the free SUMO1.

**Fig 2:**
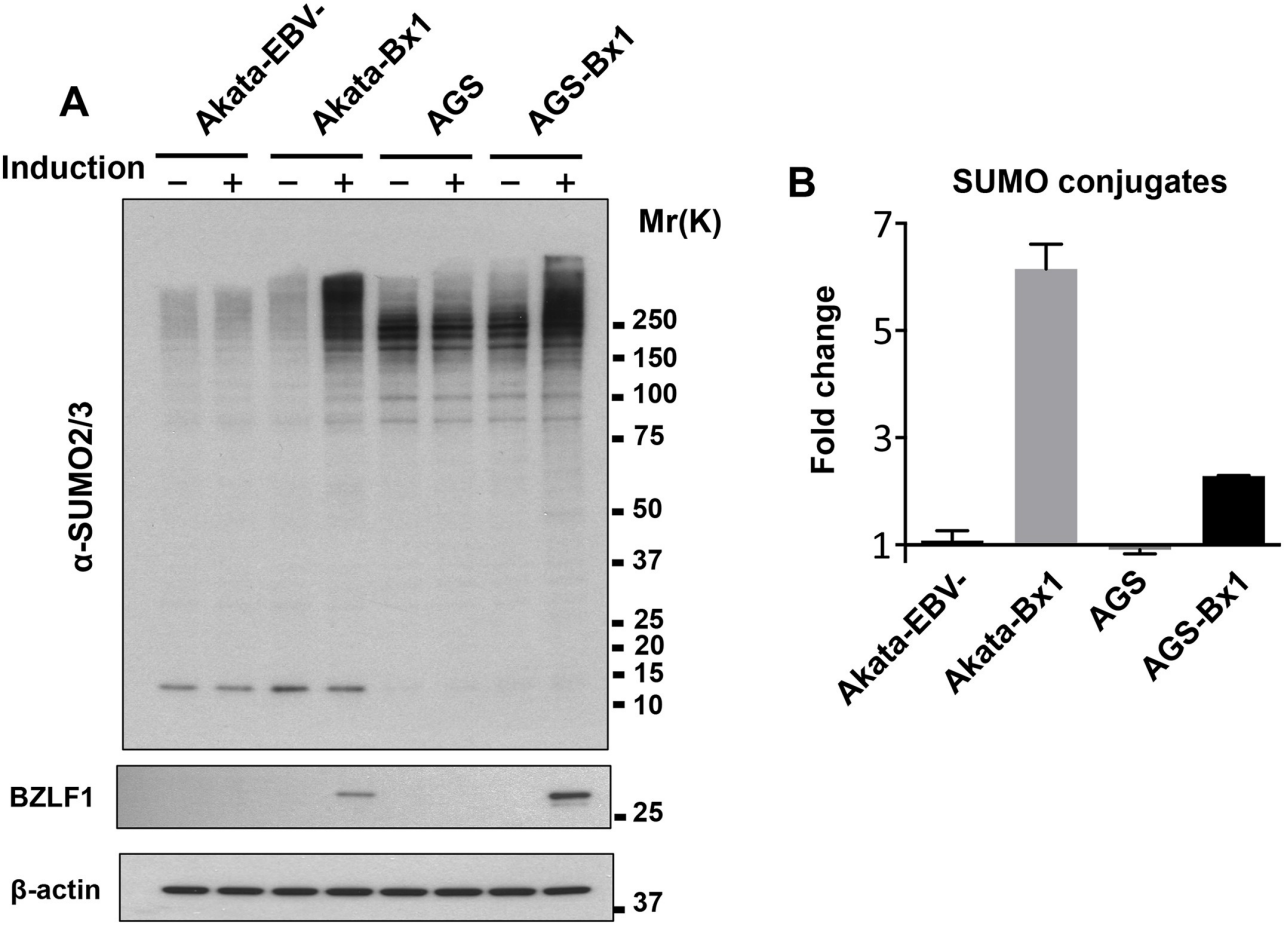
The accumulation of SUMO conjugates is dependent on virus reactivation. (**A**) Western blots of untreated and anti-IgG treated EBV negative Akata and EBV positive Akata-Bx1 and TPA/Bu treated EBV negative AGS and EBV positive AGS-Bx1 were probed with antibodies to SUMO2/3, BZLF1 and β-actin. Induction of the productive virus cycle was accompanied by accumulation of poly-SUMOylated proteins in the EBV positive cell lines. (**B**) The intensities of the SUMO2/3 reactive bands were quantified by densitometry. The average increase relative to untreated controls in two independent experiments is shown in the figure.

The abundance of SUMO conjugates was monitored in Akata-Bx1 cells following induction of the productive virus cycle by cross-linking of surface IgG. Western blots of cell lysates prepared at different times post-induction in the presence of cysteine protease inhibitors that block the activity of SUMO deconjugases were probed with antibodies specific for the EBV transactivator BZLF1, SUMO1 and SUMO2/3 (Fig 1). The activation of productive infection was confirmed by detection of a strong BZLF1 specific band starting from 8 hrs post-induction, followed by the expression of early and late viral gene products (Fig 1A and S1 Fig). Several distinct bands and a weak smear of high molecular weight species that are likely to correspond to mono-SUMOylated proteins and SUMO1 terminated poly-SUMO chains were detected by the SUMO1 specific antibody in untreated Akata-Bx1 (Fig 1B). In agreement with the notion that poly-SUMO conjugates rapidly accumulate in stressed cells [39], a significantly increased intensity of the high molecular weight species was reproducibly observed after treatment for 1 h with the anti-IgG antibody, which corresponds to time 0 of the monitoring kinetics. However, while the increase of SUMO conjugates induced by acute stress is usually transient, in productively EBV infected cells the high molecular weight species progressively accumulated, reaching a maximum 72 hrs post-induction when monitoring was ended due to increasing cell death. Re-probing the blots with antibodies to SUMO2/3 revealed a similar increase in the intensity of high molecular weight species (Fig 1C), suggesting that productive EBV infection is specifically associated with the accumulation of poly-SUMO conjugates. The specificity of the effect was confirmed by the failure to accumulate poly-SUMO conjugates in anti-IgG treated EBV negative Akata cells and TPA/Bu treated AGS cells, whereas accumulation of SUMO2/3-reactive high molecular weight species was observed in EBV positive AGS-Bx1 upon induction of the productive virus cycle by treatment with TPA/Bu (Fig 2A and 2B). A similar virus reactivation-dependent accumulation of polySUMO conjugates was observed in TPA/Bu treated B95.8 cells (S2 Fig). It is noteworthy that the virus induced effect was clearly appreciated in all EBV positive cell lines tested in spite of widely different baseline abundance of SUMO conjugates.

### The SUMO conjugation machinery is upregulated and RNF4 is post-transcriptionally downregulated in productively infected cells

**Fig 3:**
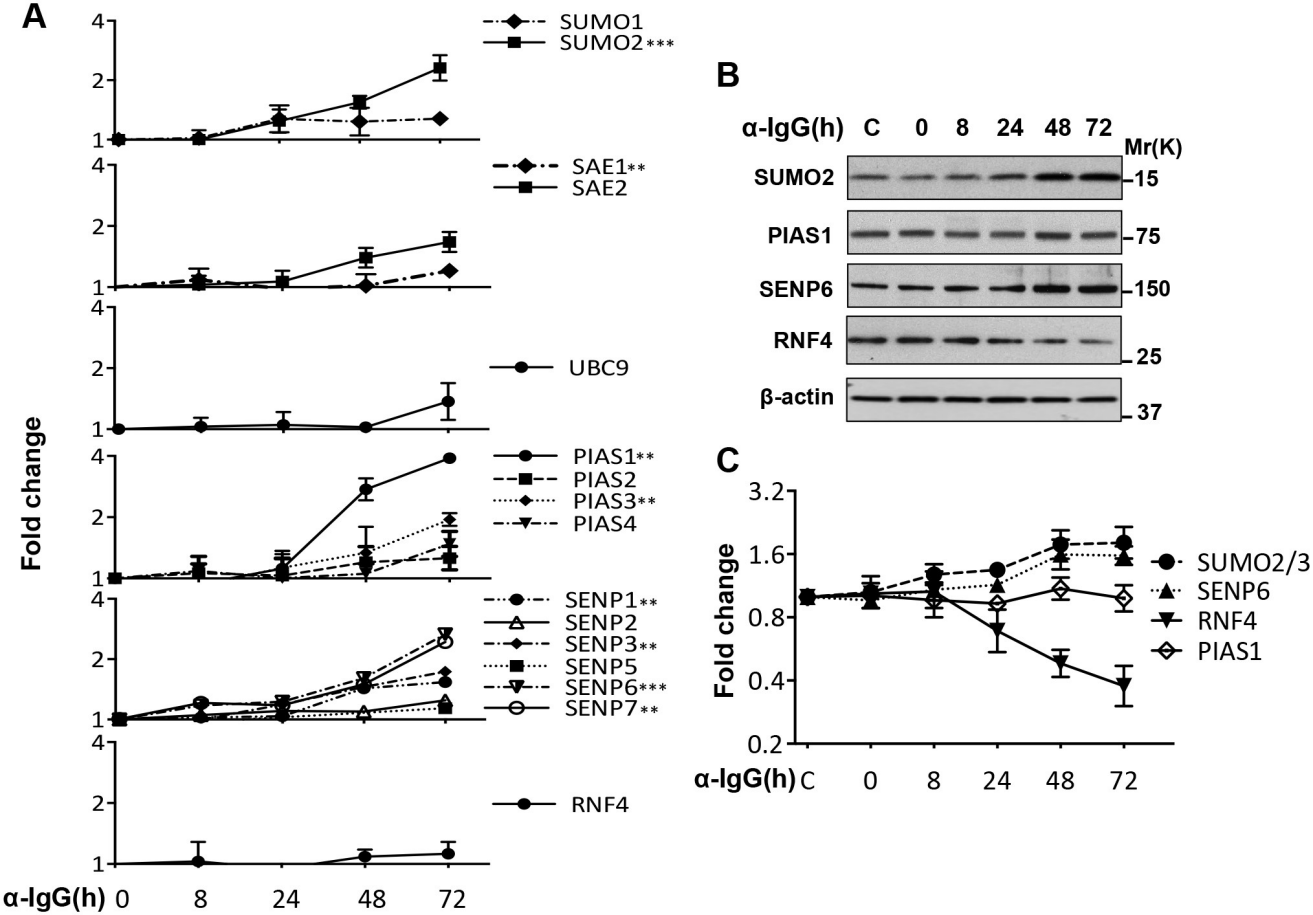
Expression of components of the SUMOylation machinery. Protein and mRNA expression was monitored over time in induced Akata-Bx1 by qPCR and western blot. (**A**) Levels of specific mRNAs measured by qPCR. The results are shown as fold change relative to the expression at time 0. The mean ± SD of three or more experiments each performed in triplicate is shown. (**B**) Representative western blots illustrating the increased expression of SUMO2, PIAS1 and SENP6 and decreased expression of RNF4. β-actin was used as loading control. One representative western blot out of three where the proteins were tested in parallel is shown. (**C**) Quantification of protein expression. The mean ± SD of three experiments is shown. Statistical significance calculated by student’s T-test is indicated as: ** = p<0.001, *** = p<0.0001.

The accumulation of poly-SUMO conjugates reflects a substantial remodeling of the SUMOylation machinery that may feature either enhanced conjugation or decreased turnover of the conjugates, due to impaired deconjugation or slowdown of ubiquitin-dependent degradation. To discriminate between these possibilities, the mRNA and protein levels of members of the SUMO conjugation cascade were monitored over time by specific qPCR and western blot (Fig 3 and S1 Table). A progressive upregulation of SUMO2 mRNA was detected starting from 8 h post-induction (Fig 3A). The SAE1 subunit of the SUMO activating enzyme, the SUMO ligase PIAS1 and PIAS3 and the SUMO specific proteases SENP1, SENP3, SENP6 and SENP7 that preferentially recognizes SUMO2/3 [40] were also significant upregulated with approximately the same kinetics, whereas the mRNA levels of SUMO1, SAE2, UBC9, SENP2, SENP5 and RNF4 were not significantly affected. The increase of SUMO2, SENP6 and to a minor extent PIAS1 mRNAs were paralleled by corresponding changes in the abundance of the encoded proteins (Fig 3B and 3C). In contrast, a progressive decrease of RNF4 was observed starting from 24–48 hrs post induction (Fig 3B and 3C).

**Fig 4:**
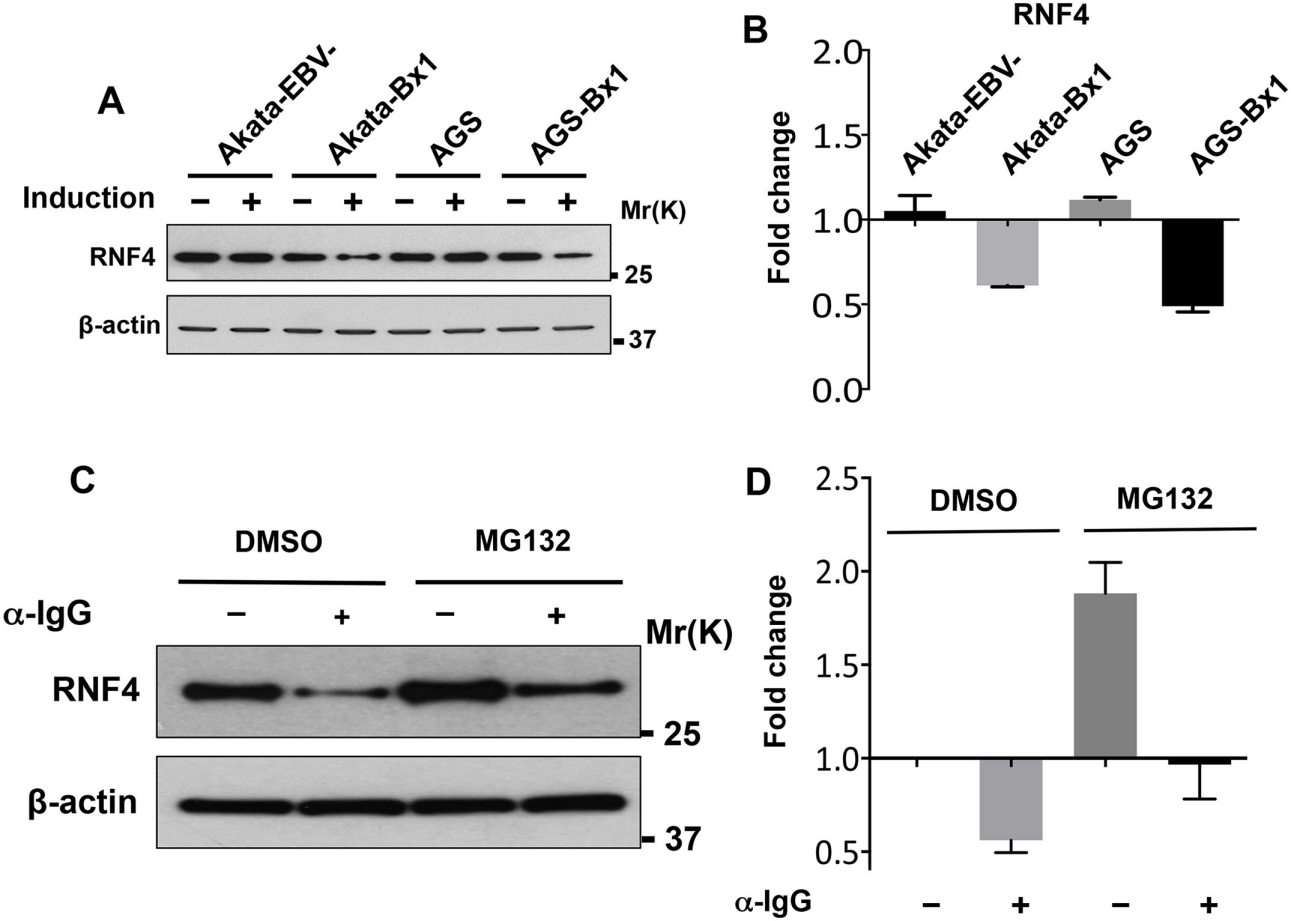
The downregulation of RNF4 is not due to increased protein turnover. The western blot shown in Fig 2 was stripped and reprobed with RNF4 specific antibodies. (**A**) RNF4 is downregulated during productive infection in the EBV positive cell lines. (**B**) The intensity of the RNF4 specific band was quantified by densitometry and fold change of was calculated relative to untreated cells. The mean ± SD of two experiments is shown. (**C**) The productive virus cycle was induced in Akata-Bx1 cells by treatment with anti-IgG for 48 hrs, and one aliquot of the cells was treated with 10 μM of the proteasome inhibitor MG132 for 6 hrs before harvesting. A significant increase of the RNF4 specific band was observed in control cells following treatment with MG132, confirming that RNF4 is a bona fide proteasome substrate. Inhibition of the proteasome did not reverse the downregulation of RNF4 in the induced cells. (**D**) The intensity of the RNF4 specific band was quantified by densitometry and fold change was calculated relative to control DMSO treated cells. The mean ± SD of three experiments is shown.

RNF4 was downregulated upon induction of the productive virus cycle in the EBV positive cell lines, Akata-Bx1 and AGS-Bx1, while this effect was not observed in the anti-IgG treated of EBV negative Akata and TPA/Bu treated AGS, suggesting that the loss of RNF4 is dependent on virus reactivation (Fig 4A and 4B). A comparable downregulation of RNF4 and accumulation of poly-SUMOylated proteins was observed upon TPA/Bu treatment in the EBV positive B95.8 cell line (S2 Fig). Furthermore, in agreement with the possibility that the downregulation may occur post-transcriptionally, the decrease of the RNF4 specific band was not reversed by treatment with the proteasome inhibitor MG132 (Fig 4C and 4D).

### The EBV miR-BHRF1-1 inhibits RNF4 expression and promotes the accumulation of SUMO conjugates

**Fig 5:**
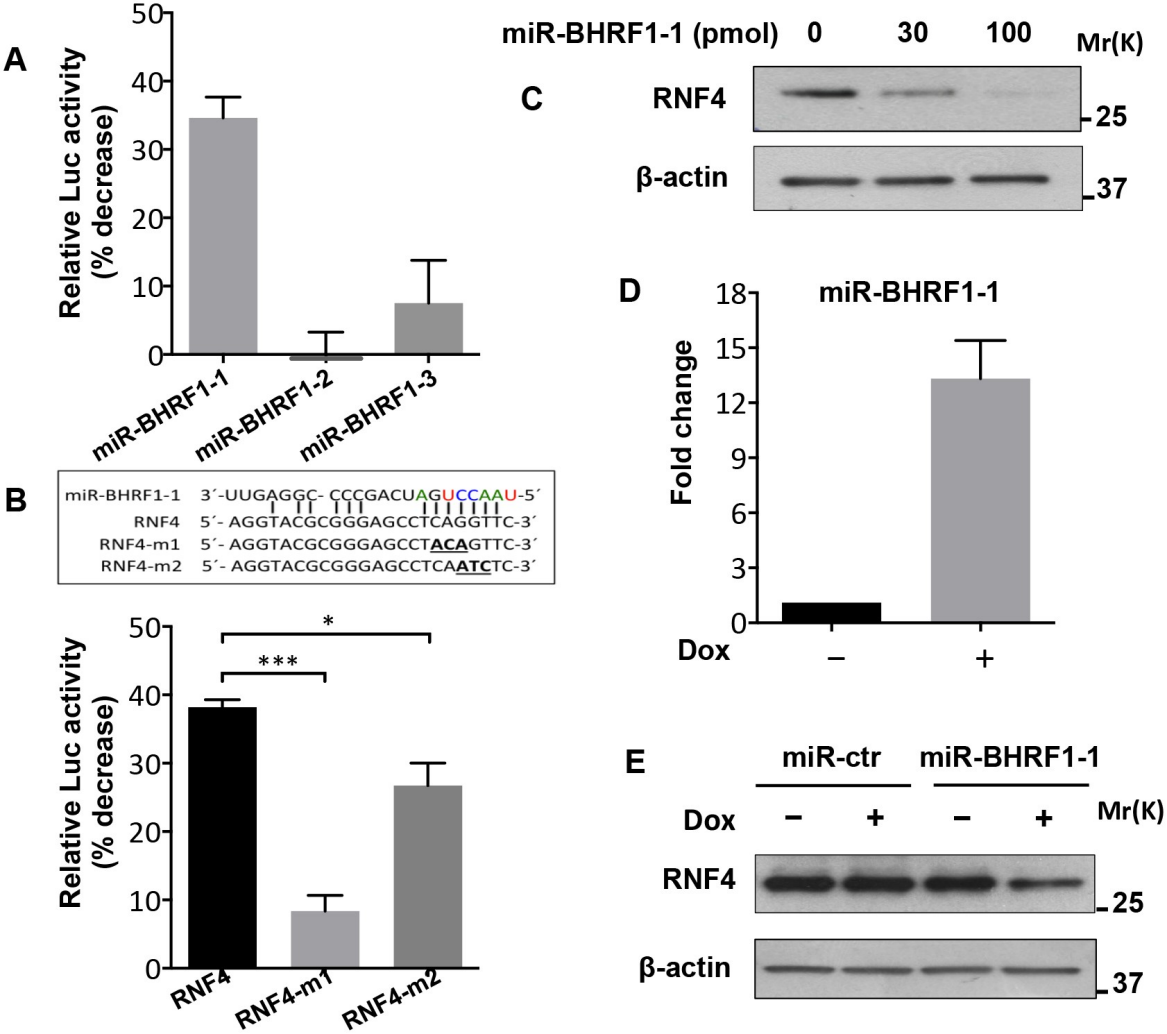
The BHRF1-1 miRNA targets RNF4. (**A**) HEK293T cells were co-transfected with an RNF4-3´UTR-LUC reporter plasmid and with plasmids expressing the indicated mature EBV miRNAs. The % luciferase activity relative to the control scrambled miRNA measured after 48h was: miR-BHRF1-1 0.65±0.03, mirBHRF1-2 1.01±0.08, miRBHRF1-3 0.92±0.06. The % decrease of luciferase activity was calculated relative to the luciferase activity of cells transfected with a control scrambled miRNA. Expression of miR-BHRF1-1 was reproducibly associated with a significant reduction of luciferase activity. The mean ± SD of three experiments is shown. (**B**) HEK293T cells were co-transfected with a plasmid expressing miR-BHRF1-1 and LUC-reporter plasmids containing wild type or mutant RNF4-3´UTR. Mutation of the miR-BHRF1-1 seed site was associated with loss of inhibition. The mean ± SD of three experiments is shown. (**C**) Representative western blot illustrating the expression of RNF4 in Akata-Bx1 cells transfected for 48 hrs with the indicated amount of a synthetic miR-BHRF1-1 oligonucleotide. (**D**) The expression of miR-BHRF1-1 in lentivirus transduced Akata-Bx1 was quantified by qPCR. A stable Akata-Bx1 subline expressing the inducible miRNA was culture in the presence or absence of doxycycline for 48 h. Mean ± SD of fold increase in three independent experiments each performed in triplicate. (**E**) Representative western blot illustrating the decrease of RNF4 in cells expressing miR-BHRF1-1. Cells transfected with a scrambled control miRNA were used as control and β-actin was used as loading control. One representative experiment out of three is shown. Statistical significance calculated by student’s T-test is indicated as: * = p<0.05, *** = p<0.0001.

MicroRNAs have emerged as an important means of viral interference with a variety of cellular functions and signaling pathways [41]. EBV infected cells express miRNAs encoded in two clusters located in the BHRF1 and BART regions of the viral genome [42]. The BHRF1 cluster is selectively upregulated during productive infection [43] and S3 Fig, suggesting that one or more of the encoded miRNAs may target RNF4. This possibility was substantiated by scanning the 3’UTR of RNF4 with six open source miRNA target prediction programs, which consistently identified high confidence target sites for the BHRF1-1 miRNA (henceforth indicated as miR-BHRF1-1, S4 Fig). The prediction was tested by measuring the effect of the miRNAs on the expression of an RNF4-3’UTR-LUC reporter. A significant reduction of luciferase activity was reproducibly observed when the reporter was co-transfected in HEK293T cells together with a plasmid expressing miR-BHRF1-1, whereas expression of miR-BHRF1-2 or miR-BHRF1-3 had only slight effects (Fig 5A). The specificity of the inhibition was confirmed by mutation of the predicted miR-BHRF1-1 seed site in the RNF4-3’UTR. A highly significant reduction of the inhibitory effect of miR-BHRF1-1 was observed when the RNF4-3’UTR seed sequence TCAGGTT was mutated to TACAGTT whereas a weaker but still significant reduction was observed with the TCAATCT mutant (Fig 5B), which may be due to different effects of the mutations on the affinity of binding to the RNF4 mRNA. The inhibitory effect of miR-BHRF1-1 was further substantiated by a dose-dependent decrease of RNF4 in Akata-Bx1 cells transfected with a corresponding synthetic oligonucleotide (Fig 5C).

To assess whether the capacity of miR-BHRF1-1 to inhibit RNF4 is also observed in a relevant cell type and under physiological levels of expression, Akata-Bx1 was stably transduced with recombinant lentiviruses expressing miR-BHRF1-1 under the control of a doxycycline-regulated promoter. Treatment of the transduced cells with doxycycline resulted in more than 10-fold increase in the levels of the miRNAs relative to untreated cells (Fig 5D), which is similar to the expression of the endogenous miRNA during productive infection (S3 Fig). Expression of miR-BHRF1-1 alone, in the absence of other lytic viral products, was sufficient for significant reduction of the RNF4 protein levels, while expression of a control scrambled miRNA had no effect (Fig 5E).

**Fig 6:**
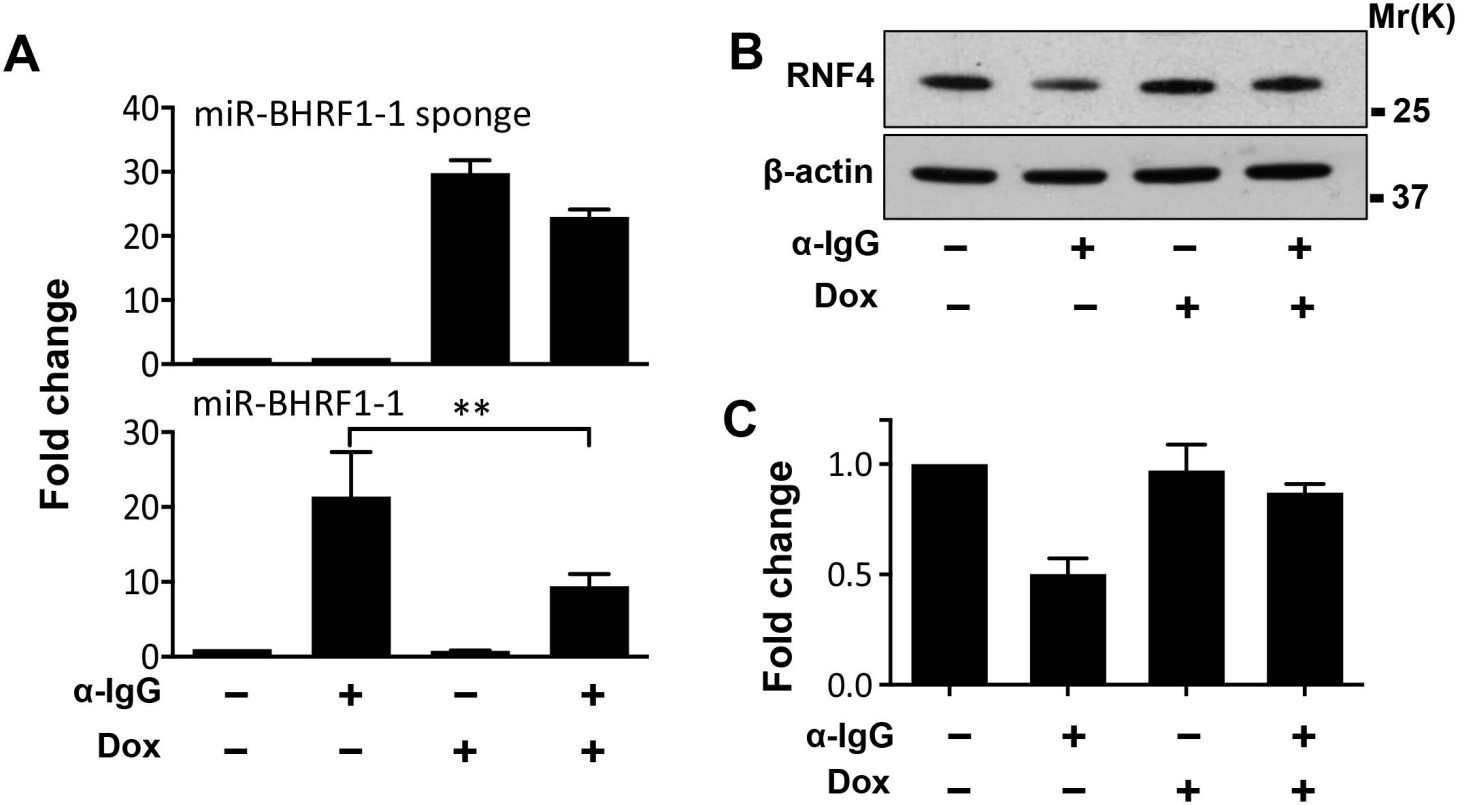
Expression of a miR-BHRF1-1 sponge prevents the downregulation of RNF4. The productive virus cycle was induced in Akata-Bx1 transduced with a recombinant lentivirus expressing a doxycycline regulated BHRF1-1 sponge. (**A**) Expression of the sponge and miR-BHRF1-1 was quantified by qPCR after 48 hrs. The upregulation of miR-BHRF1-1 was significantly reduced in cells expressing the sponge. The mean ± SD of three independent experiments is shown. (**B**) Total cell lysates were probed with a RNF4 specific antibody. The reduction of miR-BHRF1-1 in cells expressing the sponge prevented the downregulation of RNF4. One representative western blot out of three is shown. (**C**) Quantification of the RNF4 specific band in three independent experiments. Fold change was calculated relative to the expression of RNF4 in untreated Akata-Bx1.

**Fig 7:**
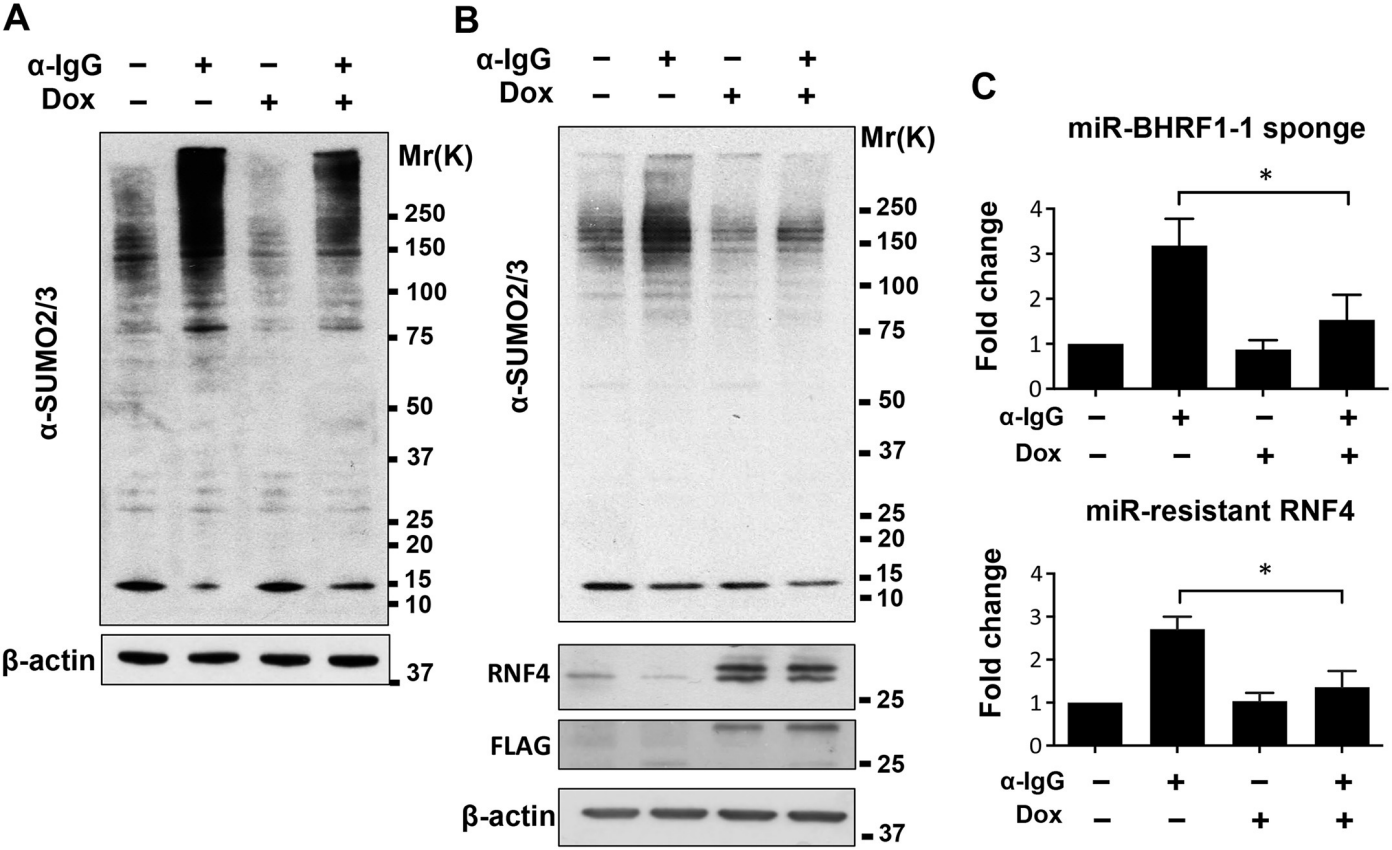
The reconstitution of RNF4 expression prevents the accumulation of poly-SUMOylated proteins during productive infection. The virus-reactivation induced downregulation of RNF4 was reversed by expression of a miR-BHRF1-1 sponge or by expression of a miR-BHRF1-1 resistant RNF4. (**A**) Expression of the sponge was associated with decreased of the SUMO conjugates. Western blots of total cell lysates harvested 48 hrs after induction were sequentially probed with antibodies specific for SUMO2/3 and β-actin. (**B**) Expression of a miR-BHRF1-1 resistant RNF4 prevents the accumulation of SUMO conjugates. Western blots of total cell lysates harvested 48 hrs after induction were probed with antibodies specific for RNF4, the FLAG tag, SUMO2/3 and β-actin. The transduced RNF4 is detected as a double band by the RNF4 specific antibody due to alternative translation that excludes the N-terminal tag. (**C**) Quantification of the western blots. The mean ± SD of two or more experiments is shown. Statistical significance calculated by student’s T-test is indicated as: * = p<0.05.

**Fig 8:**
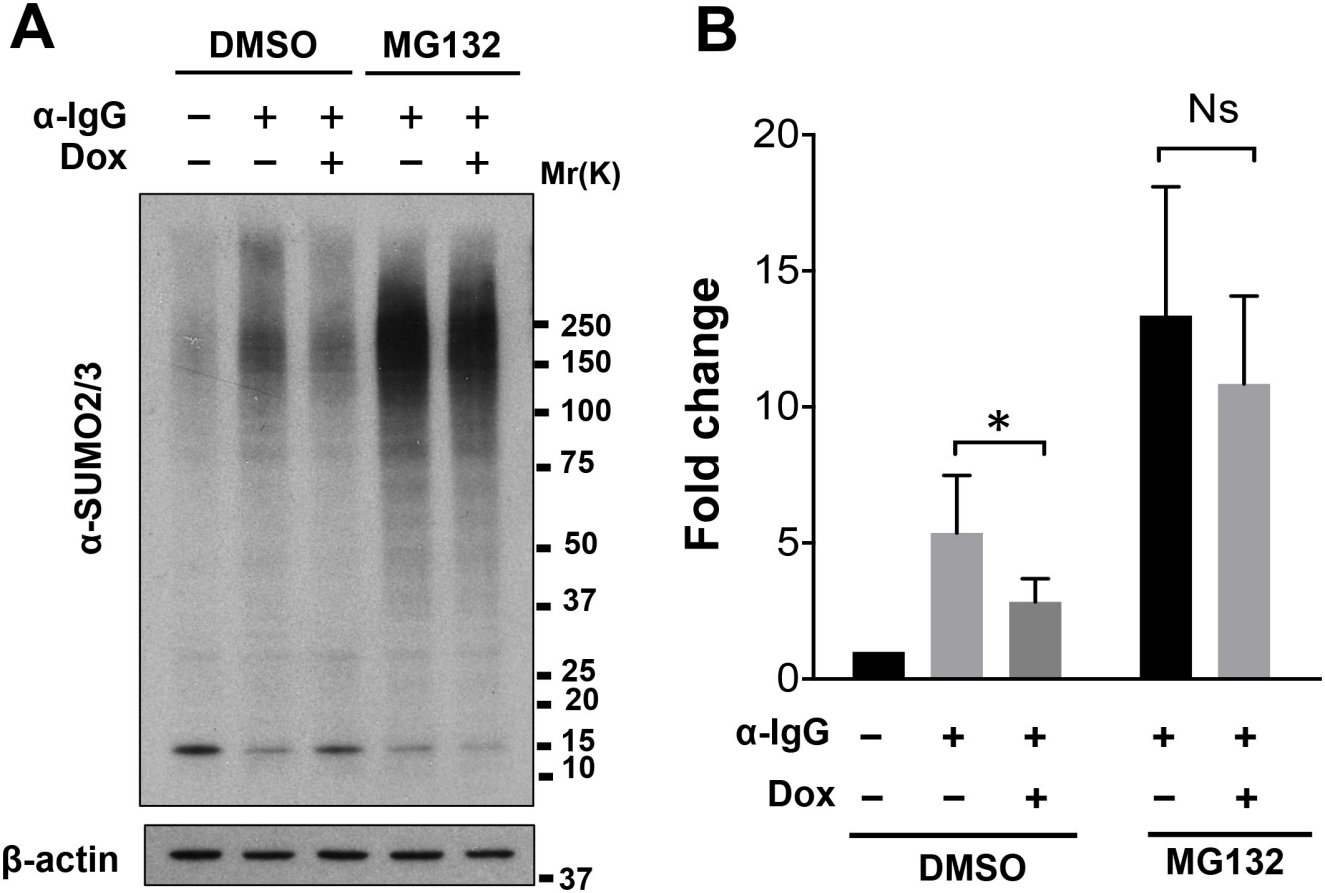
The decrease of SUMO conjugates in RNF4 reconstituted cells is due to proteasomal degradation. Untreated and α-IgG treated Akata-Bx1 were cultured for 48 hrs and treated with the proteasome inhibitor MG132 for 6 hrs before harvesting. The decrease of SUMO conjugates induced by expression of the miR-BHRF1-1 sponge was reversed by inhibition of the proteasome. (**A**) One representative western blot out of three is shown. (**B**) The intensity of the SUMO2 reactive bands was quantified by densitometry. Fold change was calculated relative to untreated Akata-Bx1. Mean ± SD of three experiments. Statistical significance calculated by student’s T-test is indicated as: * = p<0.05.

We next asked whether the upregulation of miR-BHRF1-1 is responsible for the decrease of RNF4 and accumulation of polySUMO conjugates in productively infected cells. To this end, Akata-Bx1 cells were stably transduced with a recombinant lentivirus expressing a doxycycline-regulated miR-BHRF1-1 sponge. As shown in Fig 6, expression of the sponge had no significant effect on the expression of either miR-BHRF1-1 (Fig 6A) or RNF4 (Fig 6B and 6C) in untreated Akata-Bx1, whereas it significantly reduced the upregulation of miR-BHRF1-1 and the downregulation of RNF4 in anti-IgG treated cells. In line with the possibility that the miR-BHFR1-1 mediated downregulation of RNF4 may be responsible for the accumulation of polySUMO conjugates, expression of the miR-BHRF1-1 sponge was accompanied by a significant reduction in the accumulation of polySUMO conjugates (Fig 7A and 7C). The involvement of RNF4 downregulation in the induction of this phenotype was further substantiated using a stable recombinant lentivirus transduced subline of Akata-Bx1 expressing an inducible RNF4 that lacks the 3’UTR (Fig 7B and 7C). Sustained expression of the ectopic miRNA-resistant RNF4 during productive infection was accompanied by failure to accumulate high molecular weight SUMO2/3 conjugates. In line with the capacity of the ligase to promote the proteasome-dependent degradation of poly-SUMOylated protein, the effect of RNF4 reconstitution was reversed by treatment with the proteasome inhibitor MG132 (Fig 8A and 8B). This finding supports the conclusion that the miR-BHRF1-1-mediated downregulation of RNF4 during productive EBV infection protects poly-SUMOylated proteins from proteasomal degradation.

#### Inhibition of RNF4 by miR-BHRF1-1 is required for efficient virus production

**Fig 9:**
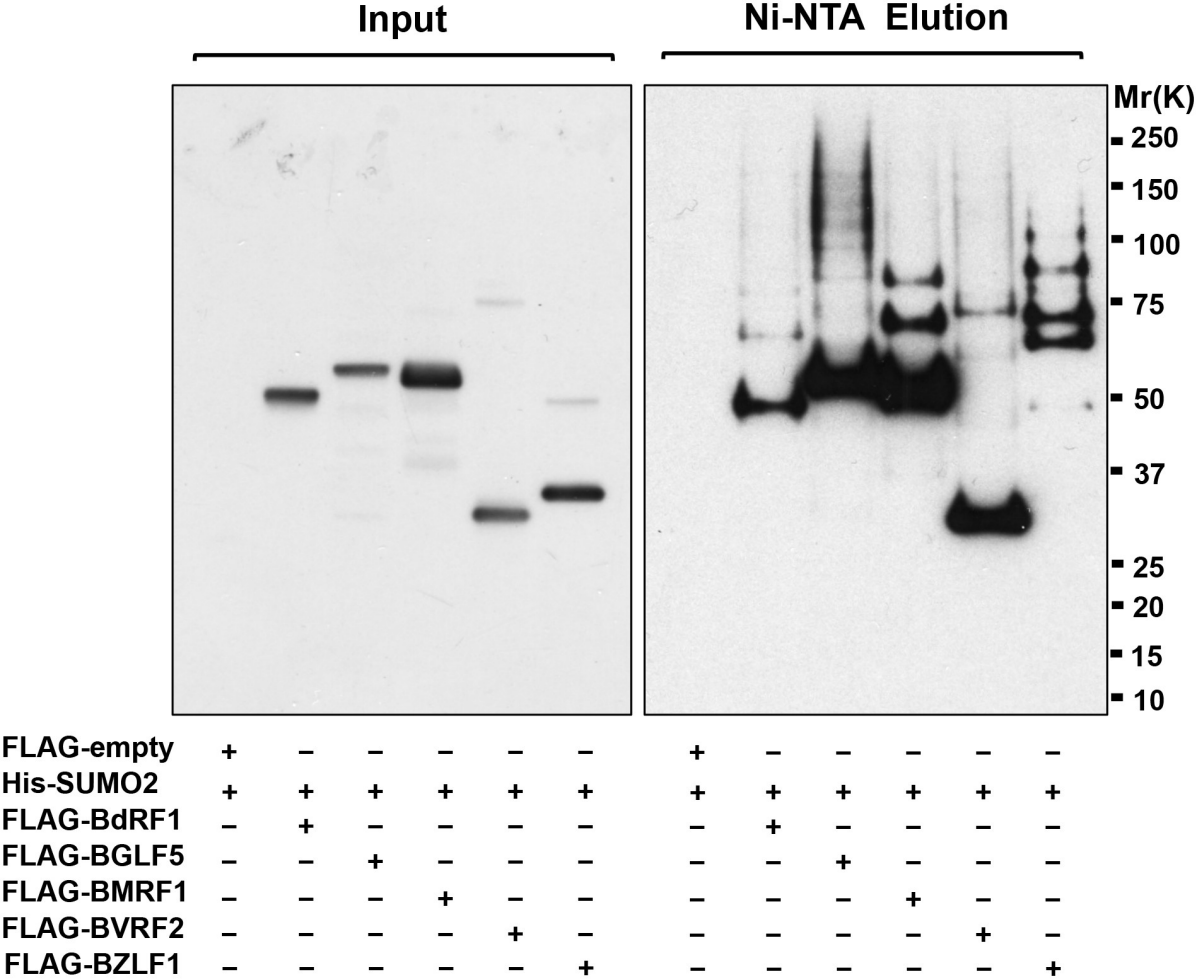
Viral proteins are bona fide SUNO substrates. Plasmids encoding FLAG-tagged version of the indicated viral proteins were co-transfected in HEK293T cells together with a plasmid expressing His-tagged SUMO2. Total cells lysates were probed with an anti-FLAG antibody (left panel). Cell pellets were lysed in denaturing buffer containing 7M urea and SUMOylated proteins were affinity purified with Ni-NTA agarose beads. Western blots were probed with the anti-FLAG antibody (right panel). High molecular weight species corresponding to poly-SUMOylated proteins were detected by the FLAG antibody. One representative experiment out of three is shown.

The accumulation of SUMOylated proteins starting from 24 h post-induction suggests that a consistent fraction of the conjugates may be viral proteins expressed during the productive cycle. To explore this possibility, we first tested whether a selection of viral proteins expressed during the productive cycle are bona fide SUMOylation substrates. Since antibodies to EBV lytic antigens suitable for direct analysis of SUMOylation are not available, FLAG-tagged versions of the coding sequences for the DNA polymerase processivity factor BMRF1 [44], the viral alkaline exonuclease BGLF5 [45], the capsid assembly scaffold protein BVRF2 [46] and the viral capsid protein BdRF1 [47] were cloned in an eukaryotic expression vectors and co-transfected in HEK293T cells together with His-tagged SUMO2 (Fig 9). A Flag-tagged version of the known SUMOylation substrate BZLF1 was included for reference. Polypeptides of the expected molecular weight were detected by an anti-FLAG antibody in western blots of total cell lysates (Fig 9A). Consistent with the described auto-proteolytic activity of BVRF2, bands of approximately 75 and 27 kDa were detected in the transfected cells [46]. SUMOylated proteins were affinity purified from cell lysates made in presence of 7M urea using Ni-NTA affinity resin and western blots were probed with the anti-FLAG antibody. High MW species of size shift corresponding to single or multiple SUMO-conjugated moieties were detected in the lysates of cells expressing the five viral proteins but not in cells transfected with the FLAG vector control, indicating that the viral proteins are bona fide SUMOylation substrates (Fig 9B).

**Fig 10:**
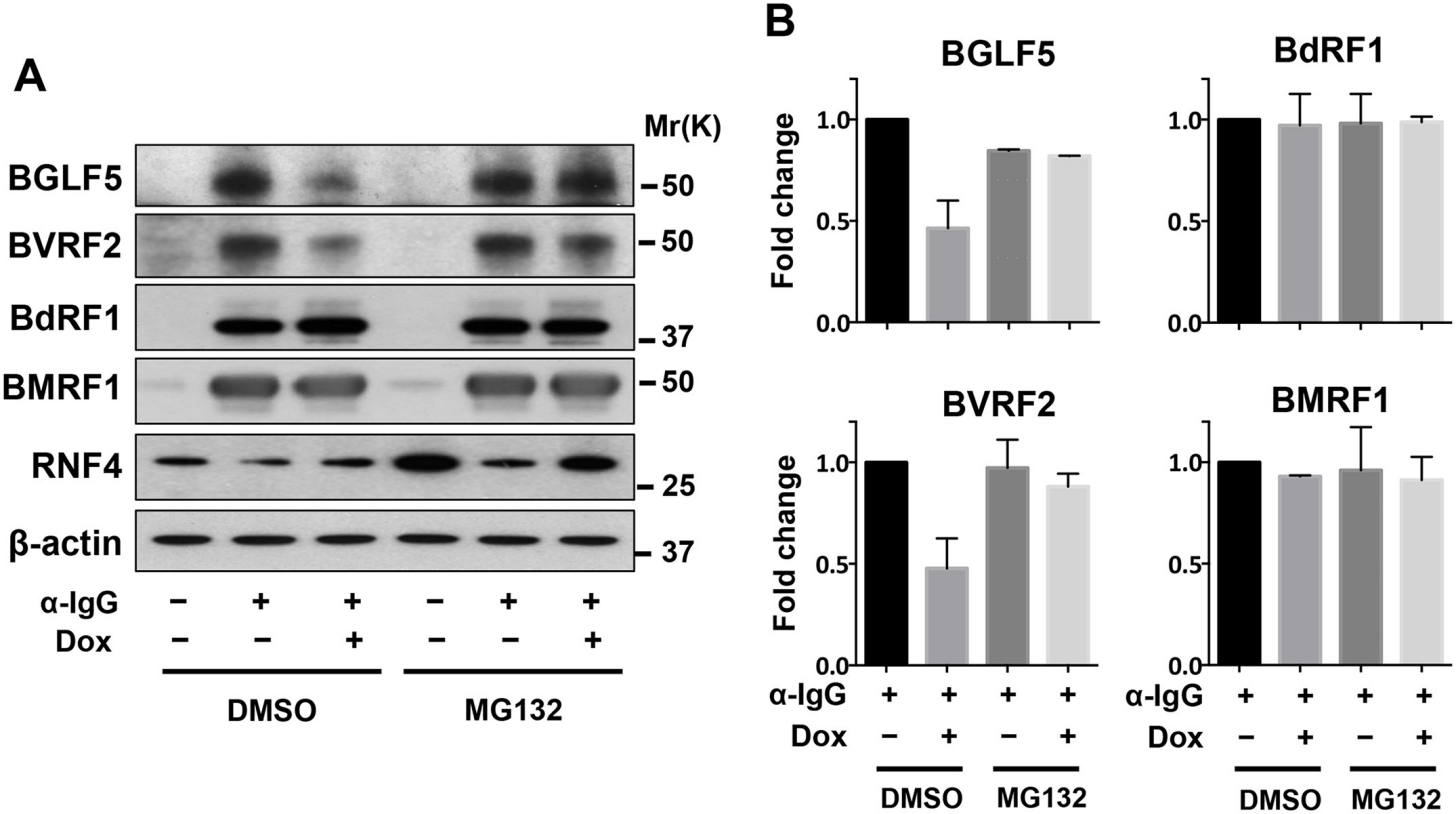
Reconstitution of RNF4 expression in productively infected cells is associated with proteasome-dependent degradation of viral proteins. (**A**) Akata-Bx1 constitutively expressing the inducible miR-BHRF1-1 sponge were treated overnight with doxycycline and then induced by cross-linking surface IgG for 48 hrs. One aliquot of the cells was treated with the proteasome inhibitor MG132 during the last 6 hrs before harvesting. Western blots of total cell lysates were probed with the indicated antibodies. The downregulation of BGLF5 and BVRF2 observed in doxycycline treated cells was reversed by treatment with MG132, indicating that the proteins are degraded by the proteasome. One representative experiment out of two is shown. (**B**) The intensities of the specific bands were quantified by densitometry and fold change was calculated relative to cells induced in the absence of doxycycline. The mean ± SD of two experiments is shown.

The parallel accumulation of SUMOylated protein and decrease of RNF4 during productive infection is consistent with a scenario where the STUbL may regulate the abundance of SUMOylated viral proteins by promoting their proteasome-dependent degradation. In order to test this possibility, the downregulation of RNF4 was reversed in anti-IgG treated Akata-Bx1 expressing the miR-BHRF1-1 sponge and the abundance of viral proteins was assessed by probing western blots of total cell lysates with specific antibodies. More than 50% reduction in the intensity of the BGLF5 and BVRF2 specific bands was detected in parallel with upregulation of RNF4 in induced cells expressing the miR-BHRF1-1 sponge, and the effect was reversed upon treatment with MG132, confirming that the decrease is due to proteasomal degradation (Fig 10A and 10B left panel). Surprisingly, we could not detect significant changes in the abundance of the BdRF1 and BMRF1 proteins.

**Fig 11:**
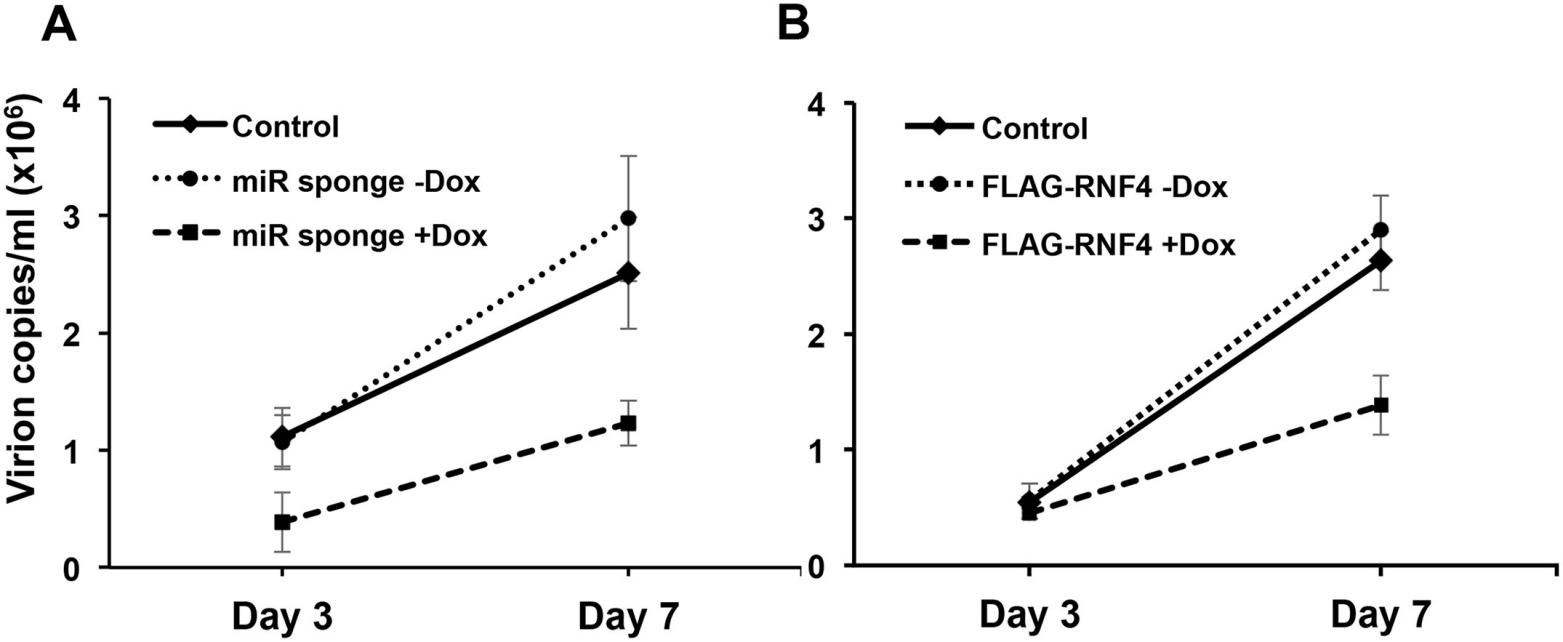
Reconstitution of RNF4 hampers the release of virus particles. The productive virus cycle was induced by anti-IgG treatment in Akata-Bx1, Akata-Bx1 expressing the inducible miR-BHRF1-1 sponge and Akata-Bx1 expressing a miR-BHRF1-1 resistant RNF4 cultured in the presence or absence of doxycycline. Intact virus particles released in the supernatants harvested after 3 and 7 days were measured by qPCR after treatment with DNase I. The amount of virus was estimated relative to a standard curve constructed by serial dilutions of a BZLF1 encoding plasmid. Reconstitution of RNF4 expression in cells expressing the miR-BHRF1-1 sponge (**A**) or miR-BHRF1-1 resistant RNF4 (**B**) was accompanied by a significantly reduced virus yield. The mean ± SD of two to three experiments is shown.

Since EBV structural proteins were found to be poly-SUMOylated, in the final set of experiments, we sought to assess the role of RNF4 downregulation in the production of infectious virus. To this end, the productive cycle was induced in Akata-Bx1, Akta-Bx1 expressing the inducible miR-BHRF1-1 sponge and Akata-Bx1 expressing the miR-resistant RNF4 in the presence or absence of doxycycline and the amount of virus particles was quantified by qPCR in culture supernatants harvested 3 and 7 days post-induction (Fig 11). In order to exclude contamination by free viral DNA or virus fragments released by damaged cells, the supernatants were treated with DNase I before DNA extraction. The amount DNase I resistant viral DNA was quantified relative to a standard curve produced using a BZLF1 encoding plasmid. In line with the finding that reconstitution of RNF4 is accompanied by the proteasome-dependent degradation of structural viral proteins, the doxycycline-induced expression of either the miR-BHRF-1-1 sponge or the miR-resistant RNF4 was accompanied by a significant decrease in the amount of released virus particles.

## Discussion

Compelling evidence supports a pivotal role of SUMO in regulating key facets of the interaction of viruses with their host cells. Here we discovered a novel function of the EBV miR-BHRF1-1 whereby, through inhibition of the SUMO-targeted ubiquitin ligase RNF4, the virus counteracts a cellular defense that operates during the late phase of productive infection.

Changes in the global levels of SUMOylation were shown to correlate with either enhanced or reduced virus yields in cells infected with different viruses [48, 49], suggesting that SUMOylation may play different roles depending on the type of virus and stage of the virus cycle that is affected. We have found that a robust accumulation of SUMO conjugates is required for efficient virus production in EBV infected cells. At least two events act synergistically in promoting the accumulation of SUMOylated proteins. First, we observed transcriptional upregulation and increased protein levels of several components of the SUMOylation machinery, which, in light of the general shutoff of cellular transcription that accompanies the productive virus cycle [50], suggests that SUMOylation is actively promoted. Second, we found that the accumulation of SUMO conjugates correlates with a progressive decrease of RNF4, resulting in very low to undetectable levels of the protein 72 hrs post-induction. Our finding that the decrease of RNF4 is dependent on the activity of a viral microRNA illustrates a new strategy by which the virus interferes with SUMO-regulated events, and highlights a previously unrecognized function of EBV miRNAs during productive infection.

The capacity of EBV miR-BHRF1-1 to specifically target RNF4 was confirmed using luciferase reporters expressing wild type and mutant RNF4 3’UTR sequences, by expression of a synthetic miR-BHRF1-1 oligonucleotide or the inducible miRNA at levels comparable to those observed during productive infection, and, most importantly, by reversal of the phenotype upon selective inhibition of the endogenous miRNA in cells expressing an inducible miR-BHFR1-1 sponge. The effect was also reproduced in different cell types, which excludes cell-specific artifacts. Since miR-BHRF1-1 is expressed at relatively low levels in latently EBV infected cells where RNF4 is clearly detected, including in the Akata-Bx1 cell line used in our experiments, the inhibition observed upon induction of the productive infection highlights the importance of the relative amounts of the miRNA in determining its functional target repertoire.

Previous work using recombinant EBV lacking the entire or individual members of the BHRF1 cluster suggested that these miRNAs contribute to B-cell transformation and may play a role in the expansion of the latent viral reservoir [51], possibly due to their capacity to promote cell proliferation and inhibit apoptosis [52]. However, although several putative targets have been identified by bioinformatics predictions and in RISC-IP studies [42], evidence for the capacity of miR-BHRF1-1 to regulate specific viral or cellular genes during latent infection is still lacking. Furthermore, cells infected with viruses lacking the BHRF1 cluster do not spontaneously enter the productive cycle as measured by the expression of immediate early and late viral genes [51], suggesting that the miRNAs are not essential for the maintenance of latency. The upregulation of the BHRF1 cluster during productive infection clearly point to a distinct role of the miRNAs in the lytic cycle. It is noteworthy that, although all the BHRF1 miRNAs are induced upon entry into lytic cycle [14, 43, 53], miR-BHRF1-1 is upregulated with a slower kinetics (S3 Fig and [43]), suggesting that it may have a specific function in the late phase of the replicative cycle when infectious virus particles are assembled. This scenario is consistent with the findings of Wahl et al. [54] who demonstrated a significant delay in the appearance of circulating viral DNA in humanized mice infected with a mutant EBV lacking the BHRF1 cluster without concomitant impairment of the oncogenic potential *in vivo*, suggesting a predominant effect on virus production and spread rather than on the proliferation of the infected cells.

Our finding that the miR-BHRF1-1 mediated inhibition of RNF4 promotes the accumulation of SUMO conjugates during the late phase of productive EBV infection suggests that the cellular ligase could have different roles in the virus cycle. RNF4 regulates the activity of PML bodies by promoting the proteasome-dependent degradation of its poly-SUMOylated components, including SP100, DAXX and PML itself [55]. PML bodies participate in the intrinsic cellular defense against virus infection and are rapidly dispersed upon induction of the productive cycle [56]. Several members of the herpesvirus family express functional homologs of RNF4, such as the immediate early proteins ICP0 of HSV1 [57] and K-Rat of KSHV [25], that promote virus production by targeting the PML bodies. EBV does not encode a functional homolog of RNF4 and it is therefore tempting to speculate that the virus may hijack the cellular protein to overcome cellular constraints that prevent virus reactivation. This putative early virus promoting effect stands in sharp contrast to the capacity of RNF4 to inhibit events that occur later during infection, as illustrated by the ubiquitination of SUMOylated BRLF1 *in vitro* and its proteasome-dependent degradation in productively infected cells, which hampers the efficiency of virus reactivation [58]. Collectively, these findings highlight the complexity of the relationship between the virus and the host cell where the same cellular function may be either harnessed or suppressed in different phases of the infection.

The concomitant downregulation of RNF4 and upregulation of several components of the SUMO conjugation machinery, together with the inhibitory effect of RNF4 on the yield of virus particles, emphasizes the importance of SUMOylation for the production of infectious virus. Given the involvement of SUMO in the regulation of nuclear transport [59, 60], SUMOylation may be required for the accumulation of viral proteins produced in the cytoplasm to the site of virus replication and assembly in the nucleus. SUMOylation may also guide the gathering of protein complexes that control the late phases of virus production, as shown for several multistep cellular processes, such as DNA repair [61]. In addition, SUMOylation may play an important role in the building of virus particles. While the assembly of virions is dependent on the local accumulation of structural proteins, high protein concentrations are likely to favor protein misfolding, with consequent aggregation and ubiquitin-dependent degradation. SUMOylation could counteract this process by promoting the solubility of aggregation-prone proteins, as illustrated by the finding that SUMOylation delays aggregation of the natively unfolded neuronal protein α-synuclein *in vitro*, whereas mutation of two lysines required for SUMOylation promotes aggregation and toxicity *in vivo* [62]. Thus, SUMOylation may be required to maintain a pool of soluble viral proteins available for incorporation into the nascent virus particles. In this attractive scenario, the increased SUMOylation capacity achieved through upregulation of the SUMO conjugation machinery, and the concomitant downregulation of RNF4, could work in concert towards the generation of infectious virus.

While a comprehensive list of the viral and cellular SUMOylation substrates in productively infected cells is beyond the scope of this report, it is remarkable that all the early and late viral proteins that were included in the study were shown to be bona fide SUMOylation substrates, suggesting that the SUMOylation of viral proteins may be more common than previously appreciated. It is also noteworthy that although putative SUMOylation sites are predicted in several EBV proteins, in particular tegument proteins and other virion components, only one of the putative substrates identified in our study, BVRF2, contains a canonical SUMO-conjugation motif. SUMOylation at non-canonical sites may be a distinctive characteristic of the viral protein or, alternatively, it may reflect the particular environment in which the modification occurs. Indeed, SUMOylation at non canonical sites has been observed also in cellular proteins, and adherence to the consensus motif was shown to drop significantly in cells exposed to heat-shock or other type of stress [63].

Collectively, our findings highlight a key role of SUMOylation in the regulation of productive EBV infection and point to the SUMO machinery as a possible target for the development of new antiviral drugs.

## Materials and methods

### Chemicals and antibodies

*N*-Ethylmaleimide (NEM, E1271), Iodoacetamide (I1149), IGEPAL CA-630 (NP40, I3021), Sodium deoxycholate monohydrate (DOC, D5670), Triton X-100 (T9284), Bovine serum albumin (BSA, A7906), Sodium dodecyl sulphate (SDS, L3771), Tween-20 (P9416), Ethylenediaminetetraacetic acid disodium salt dehydrate (EDTA-E4884), Trizma base (Tris, 93349), Sodium butyrate (Bu, B5887) and 12-O-tetradecanoylhporbol-13-acetate (TPA, 4174) were purchased from Sigma-Aldrich (St. Louis, MO, USA). Complete protease inhibitors cocktail tablets were from Roche Diagnostic (Mannheim, Germany). The following antibodies were used in immunoblot: mouse anti-βactin (AC-15, 1:20000) and mouse anti-FLAG (F-3165, 1:5000) from Sigma-Aldrich; mouse anti-EBV BZLF1 (sc-53904, 1:1000); mouse anti-EBV-EaR p85 (BORF2, sc-56979, 1:1000) from Santa Cruz Biotechnology (Santa Cruz, CA); mouse anti-SUMO2+3 (8A2, 1:3000); rabbit anti-PIAS1 (EPR2581Y, 1:10000), mouse anti-SENP6 (ab57239, 1:2000) from AbCam (Cambridge, MA, USA); mouse anti-EBV-gp220/350 (1:1000), rabbit anti-BGLF5 (1:5000), mouse anti-BMRF1 (1:15000), rabbit anti-BdRF1 (1:1000) and rabbit anti-BVRF2 (1:1000); rabbit anti-EBNA1 (1:200, purified rabbit antibody K67.3) are a gift of Dr. Jaap M. Middeldorp (CCA-VUMC, Amsterdam, The Netherlands); mouse anti-SUMO1 (21C7, 1:1000) from Invitrogen (Invitrogen, Carlsbad, CA); chicken anti-RNF4 (1:3000) was a gift of Dr. Ron T. Hay, University of Dundee, Dundee, Scotland, UK.

### Plasmids and recombinant lentiviruses

The coding sequencing of EBV BdRF1, BGLF5, BMRF1, and BVRF2 were amplified using primers: BdRF1: 5´-TGACAAGCTTATGCTATCAGGTAACGCAGGAG-3´, 5´-TCGATGAATTCTCA AGCCACGCGTTTATTCAG-3´; BGLF5 5´-TGACAAGCTTATGGCCGACGTG GATGAG-3´, 5´-TCGATGAATTCCTATGGAGTTGACTCGTCGTCG-3´; BMRF1: 5´-TGACAAGCTTATGGAAACCACTCAGACTCTCC-3´, 5´-TCGATGAATTCTT AAATGAGGGGGTTAAAGGCC-3´; BVRF2: 5´-TGACAAGCTTATGGTGCAG GCACCGTCTG-3´, 5´-TCGATGAATTCTCAAGCCACGCGTTTATTCAG-3´ and cloned in the HindIII and EcoRI sites of the eukaryotic expression plasmid p3xFLAG-CMV-10 (E7658, Sigma-Aldrich). In order to construct recombinant lentiviruses encoding the BHRF1-1 miRNA BHRF1-1 sponge and FLAG-tagged RNF4, the coding sequences of the mature miRNAs was amplified using the primers 5´-AGAGACTCGAGGCTGCCTTTGGGATGCATCACTTT-3´, 5´-AGAGAACGCGT ACGTGACATCTCGTACTGCC-3´; the BHRF1-1 sponge was annealed using the primers: 5´-TCGAGAACTCCGGGTGCGATCAGGTTAAAAAAAAACTCCGGG CGCGATCAGGTTAATATATAACTCCGGGTTGGATCAG-3´, 5´-CGCGTTAAC CTGATCCAACCCGGAGTTATATATTAACCTGATC-GCGCCCGGAGTTTTTTT TTAACCTGATCGCACCCGGAGTTC-3; the coding sequences of human RNF4 was amplified by the primers 5´-CGACCGGTATGGATTACAAGGATGACGACG ATAAGATGAGTAC AAGAAAGCGTCGT-3´, 5´-AGAGAACGCGTTCATATAT AAATGGGGTGGTA-3´. The PCR fragments were cloned in the XhoI and MluI sites of the pTRIPZ lentiviral vector for doxycycline-inducible expression (Thermo Fisher Scientific, USA). For lentivirus production, HEK293FT cells were co-transfected with the plasmids psPAX, pMD2G (Addgene, Cambridge, MA) and scrambled control/miRNA constructs using JetPEI (Polyplus, Illkirch, France) and cultured in complete medium. The medium was refreshed after one day and harvested after 2 days. After brief centrifugation, the supernatants were stored at -80°C.

### Site directed mutagenesis

A reporter plasmid (pLightSwitch-3UTR) containing the RNF4 3’UTR cloned downstream of an optimized firefly luciferase gene was purchased from SwitchGear genomics (Menlo Park, CA). The Quickchange Site-Directed Mutagenesis kit (Agilent Technologies, Santa Clara, CA) was used to make RNF4-3’UTR mutants according to the manufacturer’s protocol using primers: RNF4m1: 5´-TACGCGGGAGCCTACAGTTCTCTCAGGGGCAGCAAAG-3´; 5´-CTTTGCTGCCCCTGAGAGAACTGTAGGCTCCCGCGTA-3´; RNF4m2: 5’-AGG TACGCGGGAGCCTCAATCTCTCTCAGGGGCAGCAAAG3´; 5´-CTTTGCTGC CCCTGAGAGAGATTGAGGCTCCCGCGTACCT-3´. The mutations were confirmed by sequencing.

### Cells lines

The EBV-producing marmoset B-cell line B95.8 [64] and the EBV-negative B lymphoma line Akata [65] were cultured in RPMI-1640 medium (R8758 Sigma-Aldrich), supplemented with 10% Fetal Bovine Serum (10270, GIBCO-Invitrogen), and 10 μg/ml Ciprofloxacin (Sigma-Aldrich) (complete medium). The Akata-Bx1 cell line [66] that carries a recombinant EBV where the thymidine kinase gene was replaced by a CMV immediate-early promoter-driven green fluorescent protein was cultured in complete medium supplemented with 500 μg/ml Geneticin (GIBCO-Invitrogen). The AGS-Bx1 cell line (kindly provided by Alan Chiang, Hong Kong University, Hong Kong), and the EBV negative parental AGS were cultured in Dulbecco's Modified Eagle Medium: Nutrient Mixture F-12 (DMEM/F12) (GIBCO-Invitrogen, Carlsbad, USA) supplemented with 10% Fetal Bovine Serum. The HEK293T (ATCC CRL3216), HEK293FT (Thermo Fisher Scientific, Waltham, MA, USA) and HeLa (ATCC RR-B51S) cell lines were cultured in DMEM (Sigma-Aldrich) supplemented with 10% Fetal Bovine Serum and 2 mM L-glutamine (GIBCO-Invitrogen). For the HEK293FT cell line, the medium was also supplemented with 0.1 mM Non-Essential Amino Acids and 1 mM Sodium Pyruvate (Sigma-Aldrich). Stable lentivirus transduced Akata-Bx1 cells were selected in medium containing 1 μg/ml puromycin (Sigma-Aldrich) for more than 14 days before use in the assays. Doxycycline-regulated genes were induced by culture for 48 h or 72 h in medium containing 1 μg/ml of doxycycline (Sigma-Aldrich).

### Induction of the productive cycle and quantification of released virus

Akata-Bx1 cells were induced by incubating cells for 90 min at 37°C with rabbit polyclonal anti-human IgG (A0423, 1:100, DAKO, Glostrup Denmark) as described [67]. Induction of the productive virus cycle was monitored by GFP fluorescence or by probing western blots with antibodies to the viral transactivator BZLF1. B95.8 cells were induced by culture in medium supplemented with 10 ng/ml 12-O-tetradecanoylphobol-13-acetate and 0.2 mM sodium butyrate (TPA/Bu) for 72 hours. AGS-Bx1 cells were induced by culture in medium supplemented with 30 ng/ml TPA and 0.5 mM Bu for 48 hours. The release of infectious virus was monitored in spent culture supernatants by quantitative PCR. Briefly, supernatants collected after 3 and 7 days were cleared of cell debris by centrifugation of 5 min at 14000 rpm and treated with 20 U/ml DNase I (Promega, Madison, WI, USA) to remove free viral DNA according to the manufacturer’s protocol. DNA was isolated from the culture supernatant with DNeasy Blood & Tissue Kit (Qiagen, Hilden, Germany) and quantified by qPCR using Power SYBR Green PCR Master Mix (Applied Biosystems by Life Technologies, Woolston Warrington, UK) and primers specific for unique sequences in BZLF1: 5´-AAATTTAAGAGATCCTCGTGTAAAACAT-3´; 5´-CG CCTCCTGTTGAAGCAGAT-3´ with cycling conditions: initial 50°C 2 min, denaturation 95°C 10 min, followed by 40 cycles of 95°C for 15 sec, 60°C for 1 min. Melting curve was run by incubating the reaction mixtures at 95°C for 15 sec, 60°C for 20 sec, 95°C for 15 sec, ramping from 60°C to 95°C at a rate of 1°C/sec. The plasmid pcDNA3-3xFlag-BZLF1 was used to prepare standard curve as described [68]. The EBV copy number was calculated relative to the standard curve using the Ct value.

### Immunoblotting and affinity purification of SUMO conjugates

The cells were lysed on ice for 30 min (50 mM Tris-HCl pH 7.4, 150 mM NaCl, 1% Triton X-100, 1% sodium dodecyl sulfate, 0.5% sodium deoxycholate, 20 mM NEM, 20 mM Iodoacetamide, protease inhibitors cocktail) and protein concentration was measured with a Protein Assay kit (Bio-Rad Laboratories, Sundbyberg, Sweden). The lysate was denatured at 100°C for 10 min in NuPage loading buffer and fractionated in acrylamide Bis-Tris 4–12% gradient gel (Life Technologies Corporation, Carlsbad, USA). After transfer to PVDF membrane (Millipore Corporation, Billerica, MA, USA), the membrane was blocked in TBS containing 0.1% Tween-20 and 5% non-fat milk. Incubation with primary antibodies was carried out for 1 h at room temperature followed by incubation for 1 h with the appropriate horseradish peroxidase-conjugated secondary antibodies. The immunocomplexes were visualized by enhanced chemiluminescence (GE Healthcare Limited, UK). For affinity purification of SUMO conjugates, expression plasmids encoding FLAG-tagged version of the viral proteins were co-transfected in HEK293T cells together with a plasmid expressing His-tagged SUMO2. After 48 h, the cells were lysed in denaturing buffer containing 7 M urea, 0.1 M Na_2_HPO_4_/NaH_2_PO_4_, 0.5 M NaCl, 20 mM Imidazole. The lysates were sonicated and then mixed with 50 μl of Ni^2+^-NTA-agarose beads and incubated for 4 hrs at 4°C. After 3x washing in buffer containing 7 M urea, 20 mM Na_2_HPO_4_/NaH2PO_4_, 0.5 M NaCl, 20 mM Imidazole the bound SUMO2 conjugates were eluted in buffer containing 200 mM Imidazole, 5% SDS, 150 mM Tris-HCl pH 6.7, 30% glycerol, 720 mM β-mercaptoethanol and 0.0025% bromophenol blue as describe [69]. The eluted factions were loaded into SDS-PAGE and immunoblotted with the anti-FLAG antibody.

### Luciferase reporter assay

HEK293T cells were seeded in a 48 well plates (2x10^5^ cells/well), after 24 hours, cells were co-transfected with 50 ng pLightSwitch report vector containing RNF4 or RNF4 mutant 3’UTR fragments, 100 ng EBV miRNA-expressing plasmid pRRLSIN.PGK-PuroR (Addgene, Cambridge, MA, USA), and 1.2 ng Renilla luciferase expressing plasmid pRL-TK (Promega, Madison, WI, USA). Luciferase activity was measured 24 h post transfection using the Dual-Glo luciferase reporter assay system (Promega, Madison, WI, USA) according to the manufacturer’s instruction. The results are presented as the fold change of normalized luciferase (Firefly/Renilla).

### Quantitative RT-PCR

Messenger RNA expression of components of the SUMOylation machinery was measured by qRT-PCR. Briefly, total RNA was isolated using the RNeasy Mini Kit (Qiagen, Hilden, Germany) and reverse transcribed using a high capacity reverse transcription kit (Applied Biosystems, USA). Quantitative RT-PCR assays were setup using the Power SYBR Green PCR Master Mix (Applied Biosystems by Life Technologies, Woolston Warrington, UK) with a final primer concentration of 100 nM. The sequences of the qPCR primers are listed in ST1 Table. Stem-loop RT-qPCR assays was applied to quantify EBV encoded miRNAs based on the stem-loop reverse transcription primer method described previously [43, 70]. RNAs were extracted using the miRNeasy Mini Kit (Qiagen, Hilden, Germany) and reverse transcribed using specific reverse primers for miRNAs by a high capacity reverse transcription kit (Applied Biosystems, Foster City, CA, USA) with condition: 16°C for 30 min followed by 42°C for 30 min and 80°C for 5 min. Real-time quantitative PCR was performed using Power SYBR Green PCR Master Mix with cycling conditions: initial 50°C 2 min, denaturation 95°C 10 min, followed by 40 cycles of 95°C for 15 sec, 60°C for 1 min. Melting curves was run by incubating the reaction mixtures at 95°C for 15 sec, 60°C for 20 sec, 95°C for 15 sec, ramping from 60°C to 95°C at 1°C/sec. The values were normalized to an endogenous small nucleolar RNA control RNU48. Fold change was calculated as: Fold Change = 2^-Δ(ΔCt)^ where ΔCt = Ct target−Ct _housekeeping_ and Δ(ΔCT) = ΔCt treated− ΔCt _untreated,_ according to the Minimum Information for Publication of Quantitative Real-Time PCR Experiments (MIQE) guidelines.

## Supporting information

S1 Table List of the qPCR primers used in this work. (DOCX)

S1 Fig Expression of viral antigens in induced Akata-Bx-1. Representative western blot illustrating the kinetics of expression of immediate early, early and late proteins in productively infected cells. The productive virus cycle was induced in Akata-Bx1 by cross-linking of surface IgG and cell aliquots were collected at the indicated times. Western blots were probed with antibodies to late antigen gp350/220, early antigen BORF2, immediately early antigen BZLF1, latent protein EBNA1. β- actin was used as loading control.
(TIF)

S2 Fig Accumulation of SUMO conjugates upon EBV reactivation in B95.8 cells. Western blots of untreated and TPA/Bu treated B95.8 cells were probed with antibodies to SUMO2/3, BZLF1, RNF4 and β-actin. Induction of the productive virus cycle was accompanied by accumulation of poly-SUMOylated proteins.
(TIF)

S3 Fig The BHRF1 miRNAs are upregulated with different kinetics during productive infection. The amount of BHRF1 encoded miRNAs was quantified over time by specific qPCR in induced Akata-Bx1. The mean ± SD fold increase relative to untreated controls recorded in three independent experiments is shown. The level of expression of RNU48 was used for normalization.
(TIF)

S4 Fig EBV miR-BHRF1-1 targets the RNF4 3’UTR. **A**. The sequences of the 3′UTR of RNF4 were retrieved from the human Ensembl database [release 73] and the sequence of the 4 mature miRNAs encoded by the BHRF1 cluster (BHRF1-1, BHRF1-2, BHRF1-2* and BHRF1-3) were downloaded from the MiRBase collection. Six miRNAs-target prediction programs including miRanda, NbMICTAR, MiRTif, RNA Hybrid, PITA and Target Spy were used to predict all possible BHRF1 miRNAs target sites. The outputs of each programs including the predicted free energy of the miRNA- mRNA duplex, the scores for the context of the sites within the UTR, conservation or SVM scores are listed. The miRanda prediction was also used as the input duplex for MiRTif. A strict model for the binding sites that requires almost-perfect degree of complementarity between nucleotides 2–8 in the 5’-end of the miRNA and the 3′UTR (SEED region) was applied for miRanda and Target Spy. The scores are marked in bold when positive interactions are predicted by the default cut-off values of each program. **B**. Schematic illustration of the constructs used for expression of BRHF1 miRNAs and control random sequences.
(TIF)
