# Supplementary material for: The Epstein-Barr virus miR-BHRF1-1 targets RNF4 during productive infection to promote the accumulation of SUMO conjugates and the release of infectious virus
Source: PLoS Pathog. 2017 Apr 17;13(4):e1006338. doi: 10.1371/journal.ppat.1006338 (PMC5413087; doi:10.1371/journal.ppat.1006338)
Supplement: S1 Table — (DOCX) [file ppat.1006338.s001.docx]

**Supporting information**

**S1 Table**

| **Primer Name** | Sequence (5´to 3´) |
| --- | --- |
| **SAE1-F** | 5´-CTTCTTGTCGGCTTGAAAGG-3´ |
| **SAE1-R** | 5´-ACCATGGGGTTGAGATTCTG-3´ |
| **SAE2-F** | 5´-GACAGAGCTGACCCTGAAGC-3´ |
| **SAE2-R** | 5´-GACAGAGCTGACCCTGAAGC-3´ |
| **UBC9-F** | 5´-ACCTCATGAACTGGGAGTGC-3´ |
| **UBC9-R** | 5´-TGGTGGCGAAGATGGATAAT-3´ |
| **PIAS1-F** | 5´-CCTGGGTTTGTCCTGTCTGT-3´ |
| **PIAS1-R** | 5´-AGCTCAAGCATCCATCGACT-3´ |
| **PIAS2-F** | 5´-GGCATCAGAAATTGGGAAGA-3´ |
| **PIAS2-R** | 5´-CCTAAAGGGCACATCAAGGA-3´ |
| **PIAS3-F** | 5´-GGTCCTCTAGCTCCCATTCC-3´ |
| **PIAS3-R** | 5´-CTCAAACCGCTGGCTAGAAG-3´ |
| **PIAS4-F** | 5´-CGCTACGCCAAGAAGAACTC-3´ |
| **PIAS4-R** | 5´-CTTCTGGCTTGAGGGTCTTG-3´ |
| **SUMO1-F** | 5´-GGGGGATAAGAAGGAAGGTG-3´ |
| **SUMO1-R** | 5´-CCATTCCCAGTTCTTTTGGA-3´ |
| **SUMO2-F** | 5´-AAAAGCCCAAGGAAGGAGTC-3´ |
| **SUMO2-R** | 5´-CCAACTGTGCAGGTGTGTCT-3´ |
| **SENP1-F** | 5´-CTTGGCTCAGGCGATTTAAG-3´ |
| **SENP1-R** | 5´-CATGGCAGTGATGGTTTGAC-3´ |
| **SENP2-F** | 5´-CAGAGTCCTGCC TTCCTTTG-3´ |
| **SENP2-R** | 5´-CTCAGACCCTTGCCACTCTC-3´ |
| **SENP3-F** | 5´-TTTTGATGCCTCAGCAAGTG-3´ |
| **SENP3-R** | 5´-ATGTCAGCGAGGTGCTTTTT-3´ |
| **SENP5-F** | 5´-GGATGAACCCCTTTGTGCTA-3´ |
| **SENP5-R** | 5´-GACCATTGGCCTGACCTAA A-3´ |
| **Primer Name** | Sequence (5´to 3´) |
| **SENP6-F** | 5´-TCAAAATGGCCTTGATCCTC-3´ |
| **SENP6-R** | 5´-TTCCTTTTGCCCACAACTTC-3´ |
| **SENP7-F** | 5´-CTTTGCAGTGGGAAAGAAGC-3´ |
| **SENP7-R** | 5´-TGTCGAAGGCAATGAGTCTG-3´ |
| **RNF4-F** | 5´-CAATGAGTACAAGAAAGCGTCG-3´ |
| **RNF4-R** | 5´-AGGTCCACAATTTCATCTCCAG-3´ |
| **GAPDH-F** | 5´-TGGGCTACACTGAGCACCAG-3´ |
| **GAPDH-R** | 5´-GGGTGTCGCTGTTGAAGTCA-3´ |
| **miR-BHRF1-1F** | 5´-TTAGTAACCTGATCAGCCCCGG-3´ |
| **miR-BHRF1-1R** | 5´-GTGCAGGGTCCGAGGT-3´ |
| **miR-BHRF1-1sponge F** | 5´-CGAGAACTCCGGGTGCG-3´ |
| **miR-BHRF1-1sponge R** | 5´-CGCGTTAACCTGATCCAACC-3´ |
| **RNU48-F** | 5´-AGTGATGATGACCCCAGGTAACTCT-3´ |
| **RNU48-R** | 5´-CTGCGGTGATGGCATCAG-3´ |
