# Supplementary figures and images for: The Epstein-Barr virus miR-BHRF1-1 targets RNF4 during productive infection to promote the accumulation of SUMO conjugates and the release of infectious virus
Source: PLoS Pathog. 2017 Apr 17;13(4):e1006338. doi: 10.1371/journal.ppat.1006338 (PMC5413087; doi:10.1371/journal.ppat.1006338)

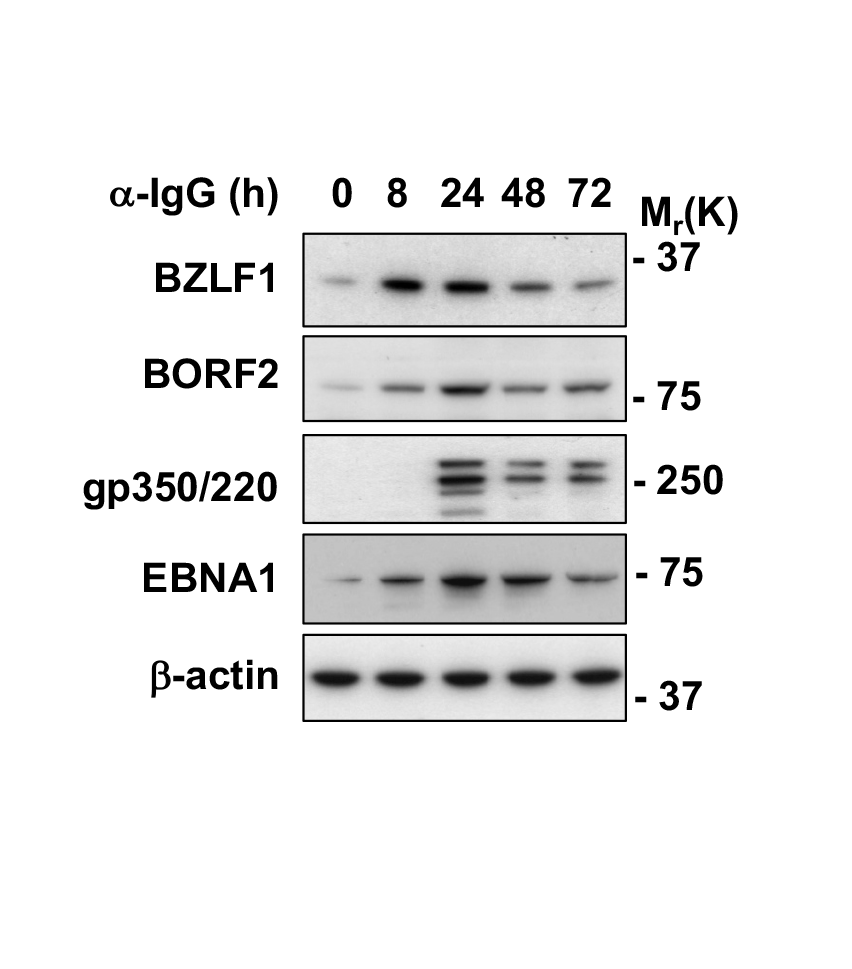

Supplement: S1 Fig — Representative western blot illustrating the kinetics of expression of immediate early, early and late proteins in productively infected cells. The productive virus cycle was induced in Akata-Bx1 by cross-linking of surface IgG and cell aliquots were collected at the indicated times. Western blots were probed with antibodies to late antigen gp350/220, early antigen BORF2, immediately early antigen BZLF1, latent protein EBNA1. β- actin was used as loading control. (TIF) [file ppat.1006338.s002.tif]

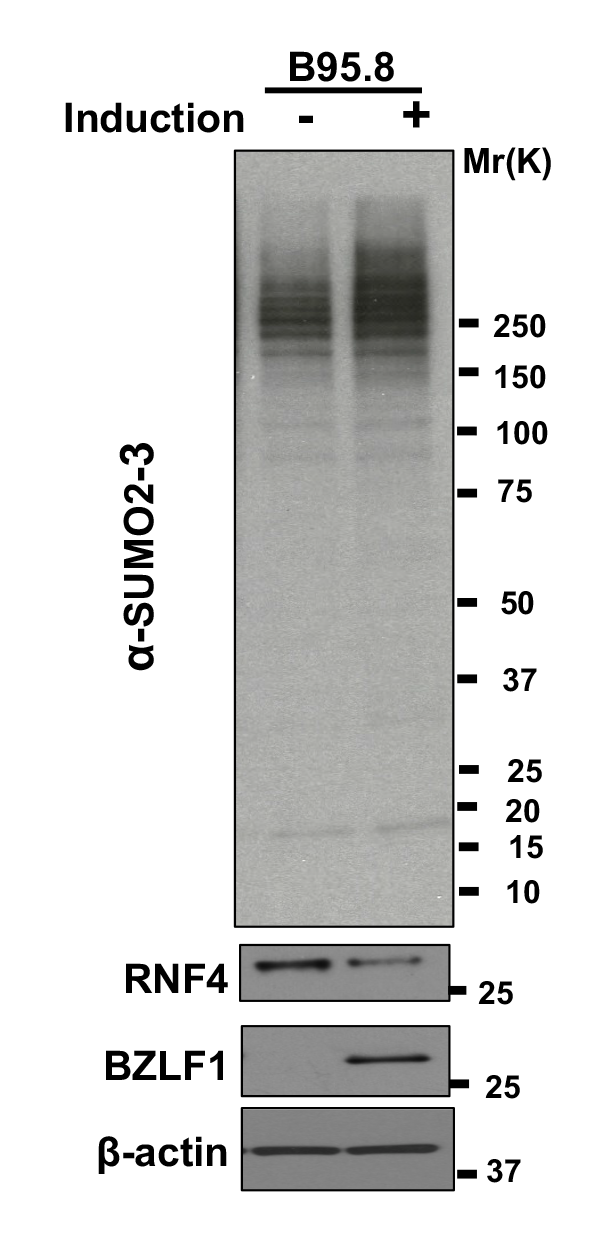

Supplement: S2 Fig — Western blots of untreated and TPA/Bu treated B95.8 cells were probed with antibodies to SUMO2/3, BZLF1, RNF4 and β-actin. Induction of the productive virus cycle was accompanied by accumulation of poly-SUMOylated proteins. (TIF) [file ppat.1006338.s003.tif]

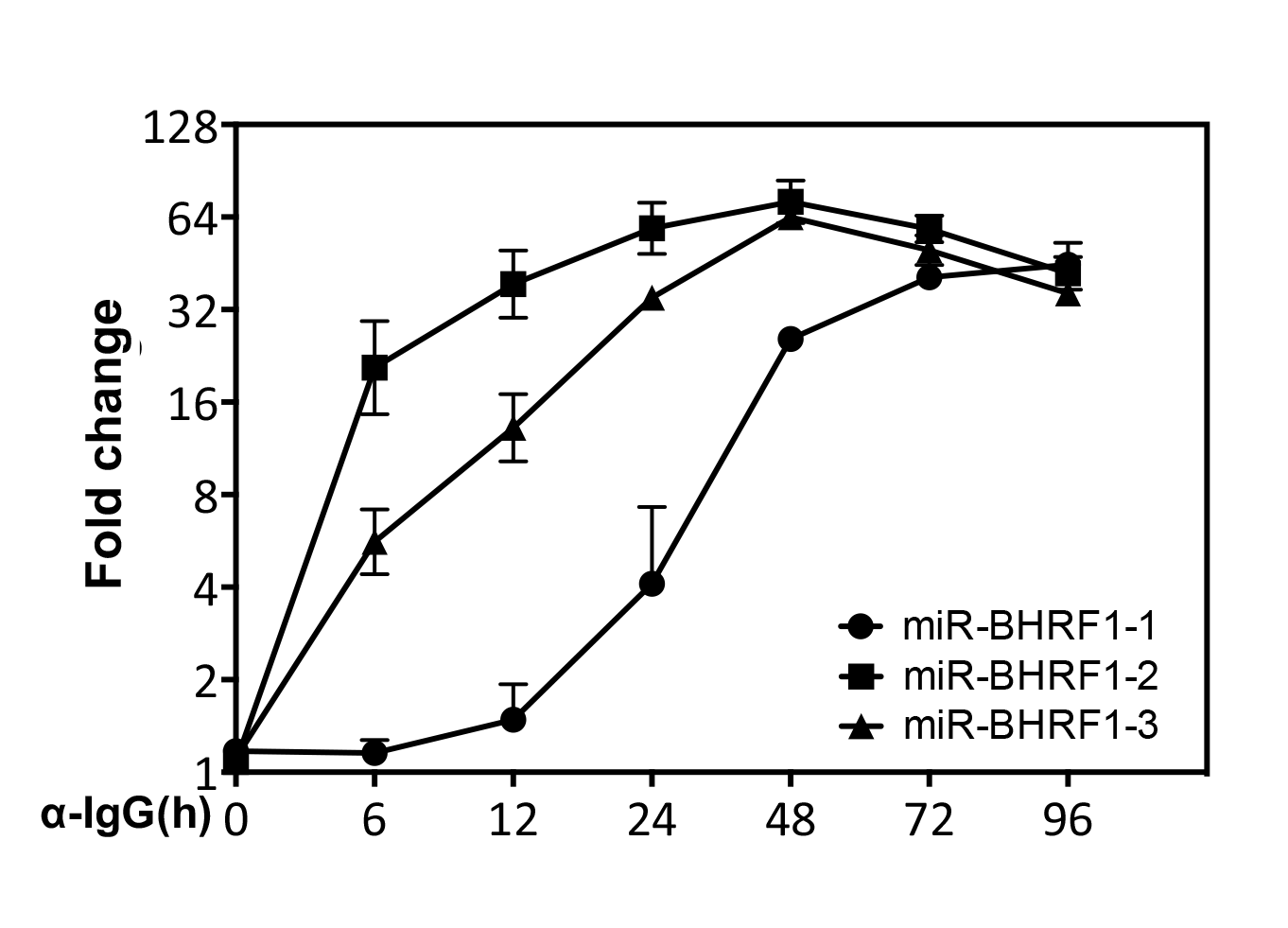

Supplement: S3 Fig — The amount of BHRF1 encoded miRNAs was quantified over time by specific qPCR in induced Akata-Bx1. The mean ± SD fold increase relative to untreated controls recorded in three independent experiments is shown. The level of expression of RNU48 was used for normalization. (TIF) [file ppat.1006338.s004.tif]

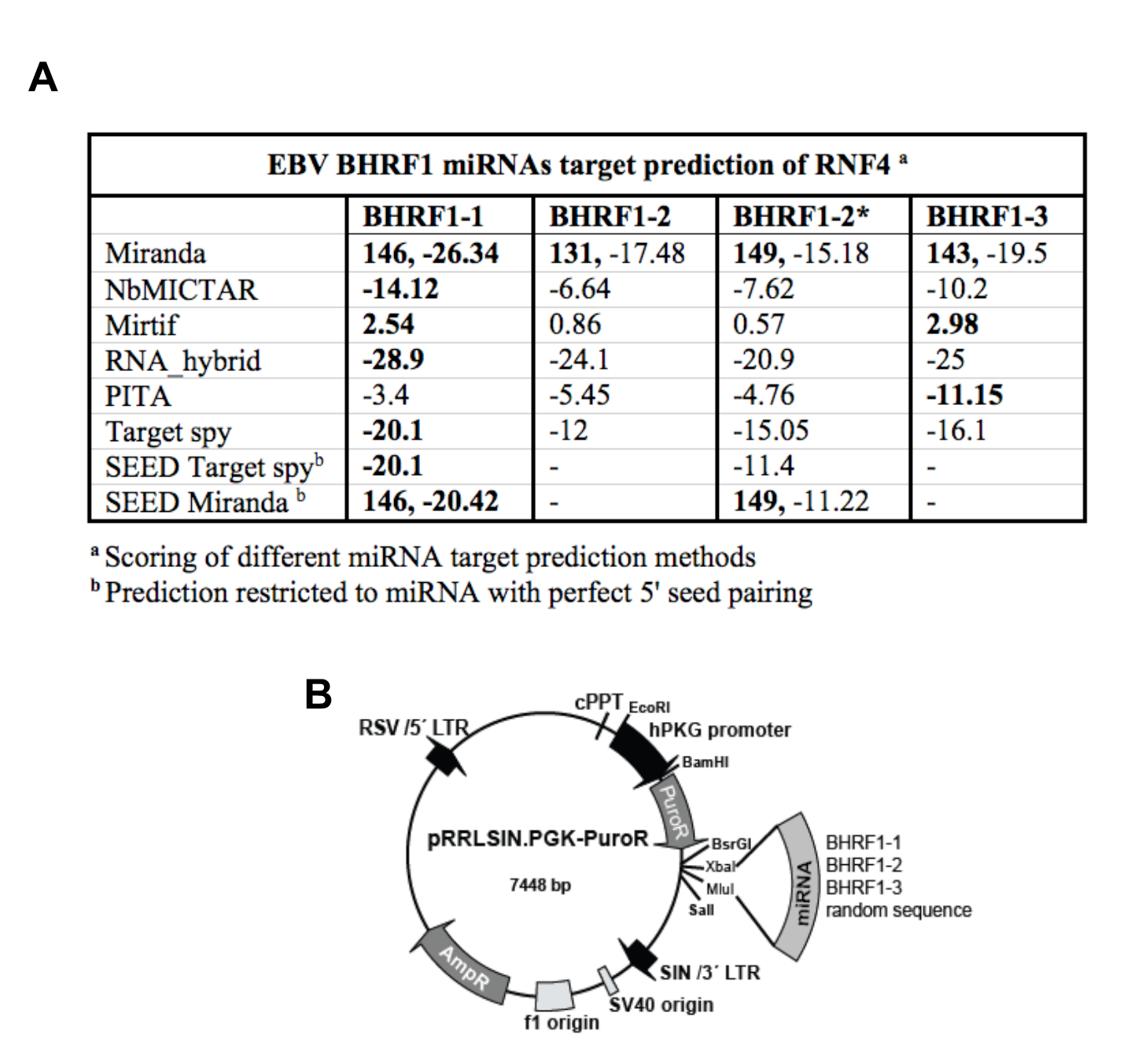

Supplement: S4 Fig — A. The sequences of the 3′UTR of RNF4 were retrieved from the human Ensembl database [release 73] and the sequence of the 4 mature miRNAs encoded by the BHRF1 cluster (BHRF1-1, BHRF1-2, BHRF1-2* and BHRF1-3) were downloaded from the MiRBase collection. Six miRNAs-target prediction programs including miRanda, NbMICTAR, MiRTif, RNA Hybrid, PITA and Target Spy were used to predict all possible BHRF1 miRNAs target sites. The outputs of each programs including the predicted free energy of the miRNA- mRNA duplex, the scores for the context of the sites within the UTR, conservation or SVM scores are listed. The miRanda prediction was also used as the input duplex for MiRTif. A strict model for the binding sites that requires almost-perfect degree of complementarity between nucleotides 2–8 in the 5’-end of the miRNA and the 3′UTR (SEED region) was applied for miRanda and Target Spy. The scores are marked in bold when positive interactions are predicted by the default cut-off values of each program. B. Schematic illustration of the constructs used for expression of BRHF1 miRNAs and control random sequences. (TIF) [file ppat.1006338.s005.tif]
